# Myosin-9 is required for lysosome-mediated nonlytic reovirus egress

**DOI:** 10.1371/journal.ppat.1013597

**Published:** 2025-10-14

**Authors:** Isabel Fernández de Castro, Martin Sachse, Gwen M. Taylor, José J. Fernández, Raquel Tenorio, Sara Y. Fernández-Sánchez, Terence S. Dermody, Cristina Risco

**Affiliations:** 1 Macromolecular Structure Department, National Center for Biotechnology, CNB-CSIC, Campus UAM, Madrid, Spain; 2 Electron Microscopy Facility, Severo Ochoa Molecular Biology Centre, CBM-CSIC, Campus UAM, Madrid, Spain; 3 Department of Pediatrics, University of Pittsburgh School of Medicine, Pittsburgh, Pennsylvania, United States of America; 4 Institute of Infection, Inflammation, and Immunity, UPMC Children’s Hospital of Pittsburgh, Pittsburgh, Pennsylvania, United States of America; 5 Subcellular Architecture Laboratory, Health Research Institute of Asturias (ISPA) and Spanish National Research Council (CINN-CSIC), Asturias, Spain; 6 Department Microbiology and Molecular Genetics, University of Pittsburgh School of Medicine, Pittsburgh, Pennsylvania, United States of America; Le Moyne College, UNITED STATES OF AMERICA

## Abstract

Mammalian orthoreoviruses (reoviruses) are nonenveloped, double-stranded RNA viruses that assemble progeny particles in cytoplasmic viral factories (VFs) and exit some types of cells using a nonlytic release mechanism. In human brain microvascular endothelial cells (HBMECs), progeny reovirus virions are selectively sorted from VFs into sorting organelles (SOs), which are derived from lysosomes. Smaller membranous carriers (MCs) bud from SOs and transport progeny virions to the plasma membrane where they are released nonlytically by fusion of MCs with the plasma membrane. To discover cellular factors required for lysosomal modification and nonlytic egress, we used mass spectrometry to identify proteins associated with lysosomes purified from uninfected and reovirus-infected HBMECs as well as virions purified from HBMECs and L929 cells, which differ in the pathways used by reovirus for egress. Network analysis of the proteomic results from HBMECs yielded an enrichment of cytoskeletal proteins centered on myosin-9. Using siRNA gene-silencing of myosin-9, pharmacological inhibition of myosin-9, super-resolution light microscopy, electron microscopy, and three-dimensional electron tomography, we found that myosin-9 acts at late stages of reovirus replication to promote viral egress. Myosin-9 associates with actin filaments attached to mature virions and mediates nonlytic egress of viral progeny from HBMECs. Our findings provide insights into the role of myosin-9 in the intracellular lysosome-mediated reovirus egress pathway and illuminate a new potential therapeutic target for viruses that use this nonlytic egress pathway.

## Author summary

Viral egress from infected cells is an important potential target for antiviral therapeutics. Mammalian reovirus can infect a wide range of cells and tissues and has been implicated in the pathogenesis of celiac disease. Early steps in reovirus infection have been characterized in detail. However, late steps in infection, including intracellular transport and nonlytic egress, are not well understood. In a previous study, we discovered a new lysosome-mediated pathway for reovirus egress. We found that reovirus uses modified lysosomes to collect mature virions on the periphery of viral replication factories, where new viruses are formed, and mediate their transport to the cell periphery for egress. In this study, we investigated the cellular factors required for the lysosome-mediated, reovirus egress pathway. We found that myosin-9, a cytoskeleton motor protein, promotes reovirus egress, participating in two steps: (i) recruitment of lysosomes to viral factories and (ii) sorting of mature virions into the modified lysosomes. Our results enhance an understanding of how viruses remodel cellular compartments to exit host cells. Most importantly, this research uncovers a new mechanism by which viruses use myosin-9 for selecting and sorting mature viruses for egress and unveils a new target for antiviral treatments.

## Introduction

Several unrelated nonenveloped viruses hijack cellular processes to exit host cells using a nonlytic pathway, while others use both lytic and nonlytic egress mechanisms. Nonlytic viral egress preserves the viability of infected cells, which can increase yields of viral progeny. Nonlytic viral egress mechanisms do not evoke inflammatory signaling associated with lytic cell death and enhance viral transmission by allowing multiple virions to be included in a single transmissible unit [1]. Some nonenveloped viruses use extracellular vesicles (EVs) to exit cells without lysis. EVs are enclosed by lipid bilayers and released from different cell types to carry proteins, nucleic acids, lipids, or metabolites [2]. EVs can bud from the plasma membrane as microvesicles or exosomes (range from 100 to 1000 nm) and are used by members of the *Reovirales*, such as rotavirus [3] and mammalian orthoreovirus (reovirus) [4]. EVs also can bud from intraluminal vesicles (ILVs) during the process of multivesicular body (MVB) formation and are released as exosomes (from 50 to 150 nm) after fusion of MVBs with the plasma membrane. The release of some members of the *Caliciviridae* (norovirus [3]), *Herpesviridae*, and *Picornaviridae* (hepatitis A virus [HAV] [5] and hepatitis E virus [HEV] [6]) families occurs using the MVB-exosomal pathway. In addition, nonlytic viral egress can be autophagosome-mediated in which single-membrane EVs are released following fusion of double-membrane autophagosomes with the plasma membrane, a mechanism termed secretory autophagy. These autophagosome-derived EVs, which range from 100 to 1000 nm, are used by coxsackievirus [7,8], poliovirus [7,9], and rhinovirus [7].

Reoviruses are nonenveloped, double-stranded RNA viruses that contain two concentric protein shells. Reovirus replication, assembly, and secondary rounds of transcription occur in large cytoplasmic structures termed viral factories (VFs) [10]. Early in infection, viral nonstructural proteins σNS and μNS remodel ER membranes to form the factory matrix [11]. Following assembly of progeny virions, reovirus can undergo either lytic or nonlytic egress depending on the cell type. Reovirus infection of HeLa, L929, and Madin-Darby canine kidney (MDCK) cells causes lysis under some circumstances. However, reovirus also can exit L929 cells in EVs using a nonlytic egress pathway [4], supporting both lytic and nonlytic release for this cell line. Likewise, reovirus uses a nonlytic egress pathway to exit human brain microvascular endothelial cells (HBMECs), but the mechanism differs from that used by the virus to exit L929 cells. Reovirus is released from polarized HBMECs predominantly from the apical surface, which allows the virus to access the bloodstream for systemic dissemination [12]. In non-polarized HBMECs, progeny virions exit cells in discrete egress zones at the basal surface [13]. This reovirus egress pathway requires the sequential action of two membranous compartments called sorting organelles (SOs) and membranous carriers (MCs). SOs are modified lysosomes recruited to the periphery of VFs during late phases of infection [13].

Reovirus infection is associated with modification of lysosome dynamics, morphology, and function. The number and size of lysosomes are increased in reovirus-infected cells, and these organelles frequently aggregate adjacent to viral factories [13]. It is possible that these lysosomal modifications allow reovirus to use this cellular organelle for egress. During reovirus infection, SOs selectively collect mature virions from VFs. Mature, genome-containing virions are attached to ﬁlaments, whereas empty capsids are neither attached to filaments nor collected into SOs. After SOs are ﬁlled with mature virions, smaller MCs bud from SOs and transport newly assembled virions to the plasma membrane for nonlytic egress [13]. Therefore, in the case of reovirus, nonlytic egress can occur by distinct pathways involving either EVs [4] or modified lysosomes [13]. However, the process by which lysosomes are modified and recruited to VFs and how mature reovirus virions are selected and transported to these SOs for extracellular release is unknown.

The presence of filaments attached to mature virions suggests a function for the cytoskeleton in lysosome-mediated reovirus egress. The cytoskeleton is a dynamic system composed of microtubules, actin filaments, and intermediate filaments [14]. These cytoskeletal components function in intracellular transport, endocytosis, and exocytosis and maintain cellular morphology [15]. Cytoskeletal components provide a dynamic scaffold for intracellular viral transport, allowing interactions of viral particles with the cellular machinery essential for internalization, disassembly, genome replication, assembly, and egress [16,17]. For some viruses, the cytoskeleton participates in the transport of progeny virions to the plasma membrane for release. For example, vaccinia virus uses microtubules and the actin cytoskeleton to promote spread of infection to neighboring cells [18]. Cytoskeletal motor proteins also are required for intracellular trafficking and dissemination of herpes simplex virus (HSV) [19,20], human immunodeficiency virus (HIV) [20,21], influenza virus [22], and severe acute respiratory syndrome coronavirus 2 (SARS-CoV-2) [23].

In this study, we identified cellular factors associated with the non-lytic, lysosome-mediated, reovirus egress pathway. Using a proteomic screen, we discovered that myosin-9, a cytoskeleton motor protein that interacts with actin to form complexes and mediate various functions within cells, is essential for nonlytic reovirus egress. In reovirus-infected HBMECs, myosin-9 associates with the filaments attached to mature virions that consist of actin. These actin-myosin filaments were detected in VFs, SOs, MCs, and released virions, suggesting a function for the filaments in reovirus release. Silencing myosin-9 expression induced aggregation and concentration of lysosomes in uninfected cells, altered lysosomal recruitment to VFs, and reduced viral egress and propagation. Inhibition of myosin-9 ATPase activity with blebbistatin did not interfere with the recruitment of modified lysosomes to VFs, but mature virions were no longer collected into SOs. These findings establish mechanistic insights into the nonlytic reovirus egress pathway, which may be applicable to other viruses that are released from cells using an analogous exit mechanism.

## Results

### Reovirus particles are detected in lysosomes isolated from reovirus-infected cells

**Fig 1:**
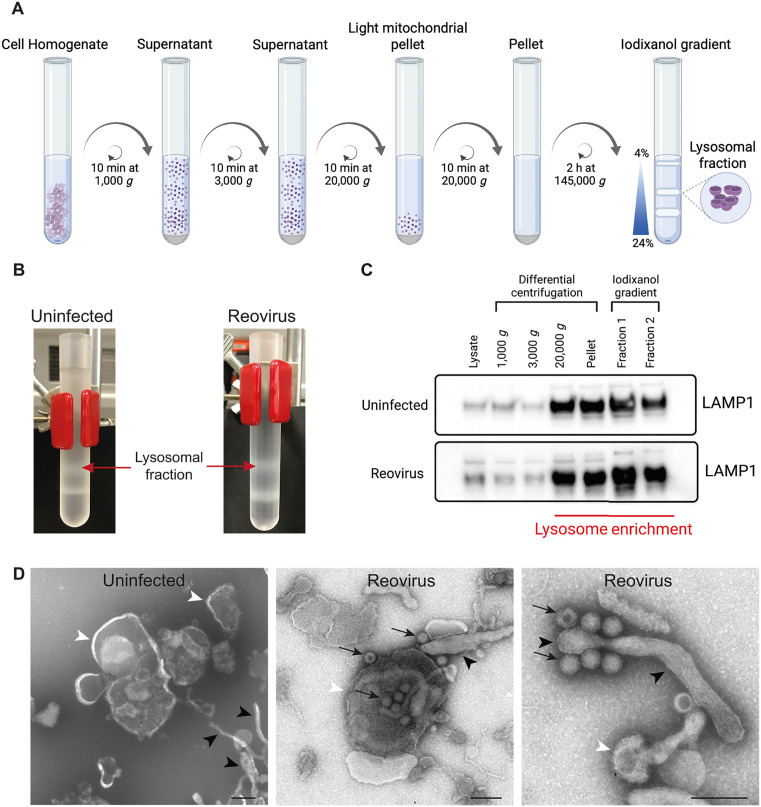
Subcellular fractionation and purification of lysosomes in uninfected and reovirus-infected cells. HBMECs were either uninfected or infected with reovirus T1LM1-P208S at an MOI of 1 PFU/cell. After incubation for 48 h, cells were scraped into homogenization buffer and processed for lysosome isolation. (A) Diagram of the workflow used for subcellular fractionation of lysosomes from HBMECs. Prepared using BioRender. Tenorio, R. (2025) https://BioRender.com/v64n455. (B) Representative photographs of iodixanol-density gradients showing mitochondria and lysosome-enriched fractions from uninfected and reovirus-infected HBMECs. The lysosomal fraction is indicated. (C) Immunoblot of cell lysates, subcellular fractionation intermediates, and individual fractions (5 µg protein) from uninfected and reovirus-infected cells show enrichment of lysosomal protein LAMP1. (D) Negative-stain TEM of purified lysosomes. Spherical and tubular organelles (white and black arrowheads, respectively) compatible with lysosomes are shown on the left (uninfected). Reovirus particles (arrows) associated with lysosome-like organelles (white arrowheads labeling spherical compartments or black arrowheads labeling tubular structures) are shown in the middle and right (reovirus). Scale bars, 200 nm.

During reovirus infection, lysosomes are modified and recruited to the periphery of VFs, where they become SOs [13]. To define the composition of these modified lysosomes, we purified lysosomes from uninfected and reovirus-infected HBMECs by cell homogenization and differential centrifugation (Fig 1A). Using iodixanol-density gradients (4–24%) and ultracentrifugation, we isolated the lysosomal fraction (Fig 1B). Lysosomes were enriched in the F1 fraction, as characterized by lysosomal membrane protein LAMP1 (Fig 1C). Using negative-stain transmission electron microscopy (TEM) of LAMP1-positive fractions, spherical and tubular organelles with electron-dense material, characteristic of lysosomes, were observed in uninfected cells (Fig 1D, white and black arrowheads). In reovirus-infected cells, intact viral particles (Fig 1D, black arrows in middle and right panels) were detected in the LAMP1-positive fractions associated with lysosome-like spherical and tubular organelles (white and black arrowheads). The association of reovirus particles with purified lysosomes suggests that the lysosomal fractions include SOs.

**Fig 2:**
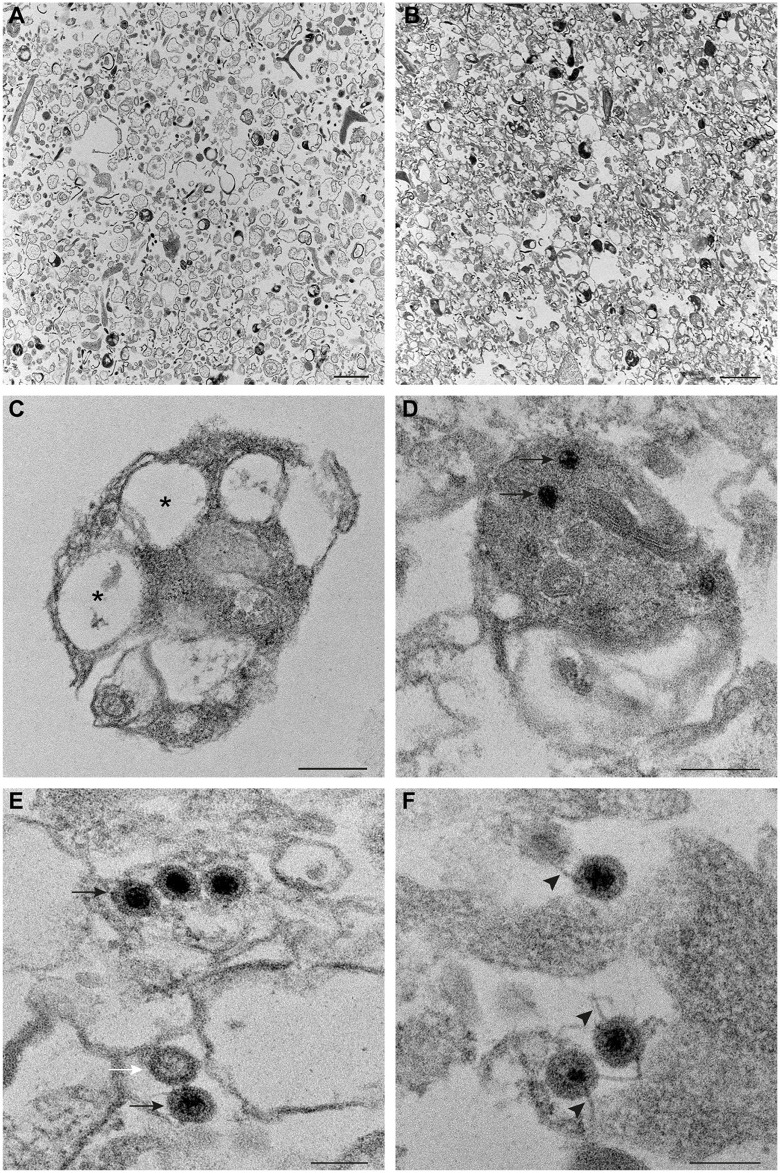
Electron microscopy of ultrathin sections of lysosomes purified from mock- and reovirus-infected cells. HBMECs were either uninfected or infected with reovirus T1LM1-P208S at an MOI of 1 PFU/cell and incubated for 48 h. Lysosomes were isolated from cells and imaged using TEM. (A and B) Low-magnification images of lysosomes purified from uninfected (A) and reovirus-infected (B) cells. Scale bars, 1 µm. (C and D) Higher-magnification images show the morphology of purified lysosomes. Vacuoles (asterisks) within electron-dense material are seen inside a lysosome isolated from uninfected cells (C). Viral particles (arrows) contained within a lysosome isolated from reovirus-infected cells are shown in D. Scale bars, 200 nm. (E and F) Viral particles present in lysosomes purified from reovirus-infected cells. Viral particles (black arrows) and an empty capsid (white arrow) are associated with a membranous structure (E). Filaments (arrowheads) attached to viral particles are seen in F. Scale bars, 100 nm.

To study details of the internal structure of the purified lysosomes, ultrathin sections were imaged using TEM. Electron-dense cellular organelles compatible with lysosomes were observed in uninfected and reovirus-infected lysosomal fractions (Fig 2A and 2B, respectively). Lysosomes purified from uninfected cells contained vacuoles (asterisks) and electron-dense material (Fig 2C), while those purified from infected cells contained viral particles (Fig 2D, arrows). In the lysosomal fraction from infected cells, mature virions (black arrows) and empty capsids (white arrow) were associated with membranes (Fig 2E). Mature virions also were attached to filaments (arrowheads) (Fig 2F). Therefore, the lysosomal fraction from infected HBMECs contained intact reovirus particles attached to membranes and filaments, suggesting that these samples incorporate host factors required for lysosome modification and release of reovirus virions using the intracellular SO-mediated, nonlytic egress pathway.

### Proteomic analysis of lysosomes purified from reovirus-infected HBMECs identifies cellular proteins associated with the ER and cytoskeleton

**Fig 3:**
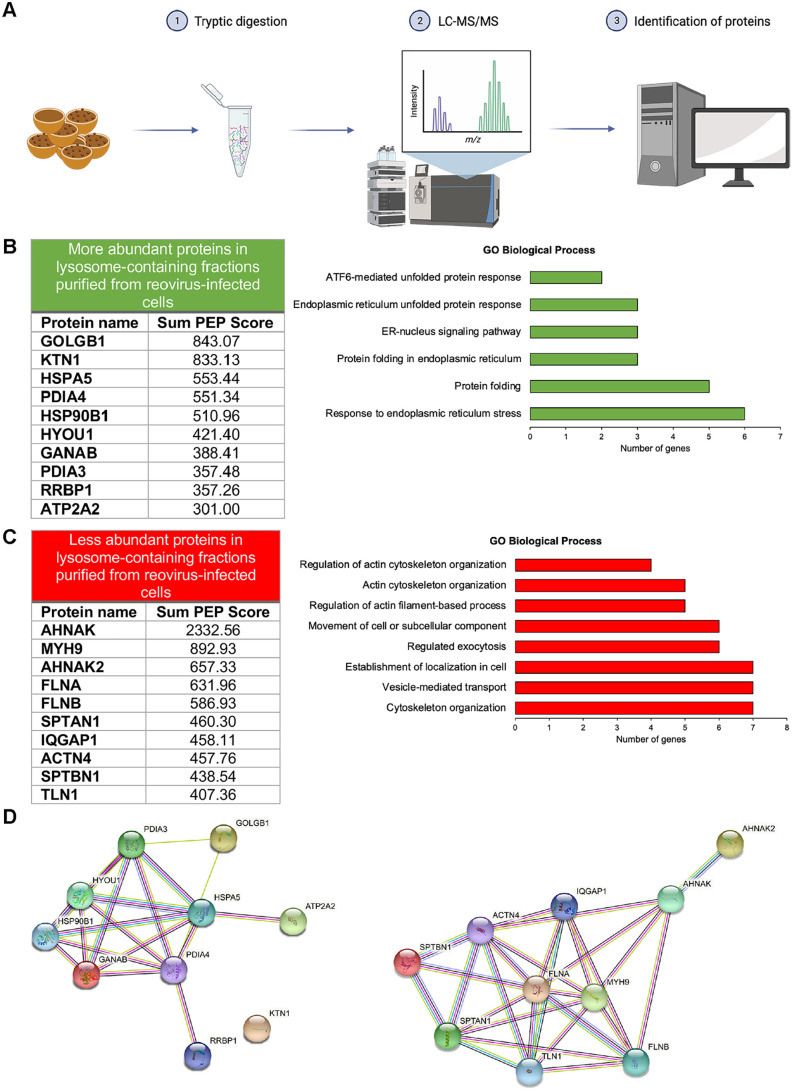
Comparative proteomic analysis reveals proteins associated with lysosome-containing fractions purified from reovirus-infected cells. Lysosomes were isolated from uninfected or reovirus-infected HBMECs by iodixanol-gradient ultracentrifugation. Proteins were extracted, digested with trypsin, and analyzed by liquid chromatography and mass spectrometry. Proteins were identified using the MASCOT database. Potential candidates with a Q-value < 0.01 and at least two peptides identified were selected for analysis. (A) Overview of the experiment workflow. Prepared using BioRender. Tenorio, R. (2025) https://BioRender.com/y38x469. (B and C) The top 10 more or less abundant candidates, respectively, identified in lysosomes purified from infected cells were compared with the candidates identified from uninfected cells. The Sum PEP Score that corresponds to the score calculated based on the posterior error probability (PEP) values of the peptide spectrum matches (PSM) is shown and calculated as the negative logarithm of the PEP values of the connected PSM. The graphs show the biological processes using Gene Ontology (GO). (D) Functional protein-protein interaction networks of the more or less abundant candidates identified using STRING analyses. See S1 Table for protein names and UniProt accession numbers.

To identify host factors associated with lysosomal fractions during reovirus infection of HBMECs, we used liquid chromatography and mass spectrometry (Fig 3A). By comparing cellular proteins identified in lysosome-containing fractions purified from uninfected and reovirus-infected cells, we identified proteins that were significantly more and less abundant in lysosome-containing fractions from infected cells (Fig 3B and 3C). Proteins with Q-values < 0.01 and at least two peptides identified were ranked according to the highest Sum PEP Score values [24]. We analyzed the top 10 protein candidates that were more or less abundant in the lysosomal fraction purified from reovirus-infected cells using Gene Ontology (GO) terms to identify biological processes and STRING analysis (Fig 3D) to identify protein-protein interactions. The top ten protein candidates that were more abundant in lysosome-containing fractions purified from reovirus-infected cells include proteins associated with the ER (Fig 3B). The top ten less abundant proteins in lysosome-containing fractions purified from reovirus-infected cells were related to the cytoskeleton (Fig 3C). We quantified myosin-9 and actin expression in uninfected and reovirus-infected HBMECs by immunoblotting using antibodies specific for myosin-9 and actin (S1 Fig). Expression of both myosin-9 and actin were comparable in both uninfected and infected cells, suggesting that their lower abundance in lysosome-containing fractions purified from infected cells is due to a change in distribution rather than a change in expression. Together, these data indicate that a unique group of host proteins are associated with lysosomes during reovirus infection, suggesting that these proteins function in SO formation and viral egress.

### Myosin-9 is associated with purified virions from HBMECs

**Fig 4:**
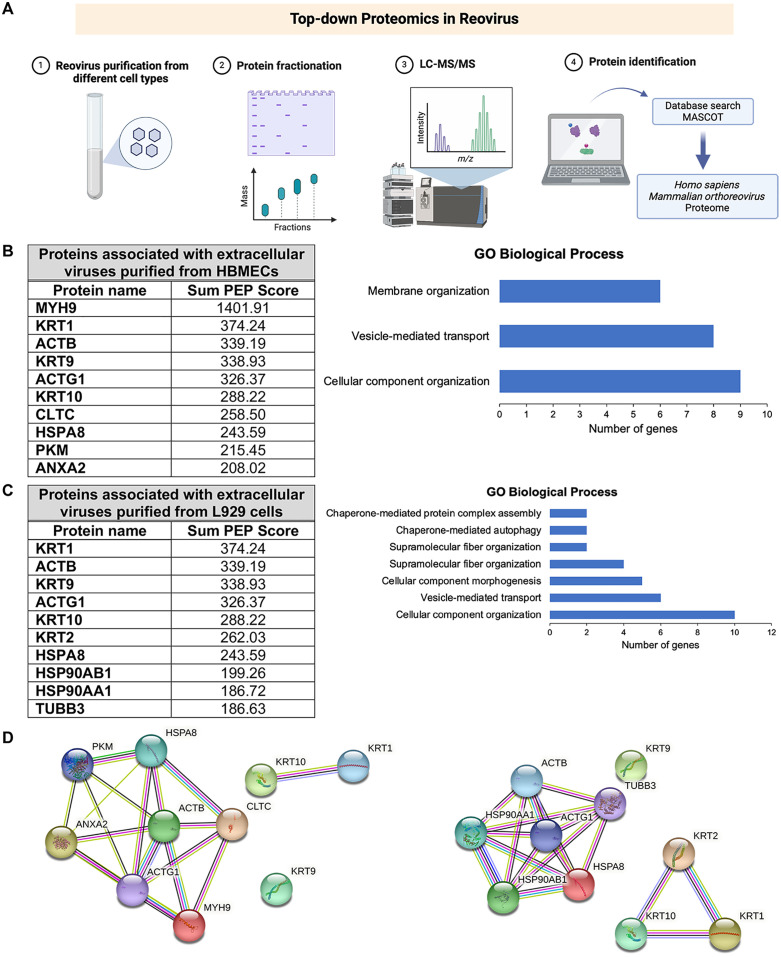
Proteomic analysis of extracellular virions purified from HBMECs and L929 cells identifies myosin-9 as a potential cellular mediator of reovirus nonlytic egress. Extracellular virions were purified from culture supernatants from HBMECs and L929 cells by iodixanol-gradient ultracentrifugation. Virions were digested with trypsin and analyzed by liquid chromatography and mass spectrometry. Proteins were identified using the MASCOT database. Potential candidates with a Q-value < 0.01 and at least two peptides identified in all three replicates were selected for analysis. (A) Overview of the proteomic analysis workflow. Prepared using BioRender. Tenorio, R. (2025) https://BioRender.com/w10l975. (B and C) The top 10 candidates from the proteomic analysis of reovirus virions purified from HBMECs and L929 cells were ranked by their Sum PEP Scores. The graphs show the biological processes using Gene Ontology (GO). (D) Functional protein-protein interaction networks of the protein candidates identified using STRING analyses. See S1 Table for protein names and UniProt accession numbers.

Reovirus uses a lytic or nonlytic egress pathway depending on the infected cell type. In L929 cells, reovirus release is associated with lysis or occurs nonlytically within EVs [4], while in HBMECs, reovirus uses a nonlytic, lysosome-mediated egress mechanism [12,13]. Mature reovirus virions in HBMECs are associated with filaments in VFs, SOs, and MCs, suggesting that filaments function in transporting mature virions through the HBMEC nonlytic egress pathway. To identify cellular factors associated with mature virions that may function in nonlytic reovirus egress, we purified intracellular and extracellular virions from HBMECs and L929 cells, which have similar replication kinetics [13,25–28]. Reovirus proteins were fractionated and analyzed by liquid chromatography coupled with mass spectrometry, and protein candidates were identified using the MASCOT database (Fig 4A). Candidates with Q-values < 0.01 and at least two peptides identified were ranked according to the highest Sum PEP Score values. The top ten candidates associated with extracellular virion-containing fractions purified from both HBMECs and L929 cells include cytoskeletal proteins (Fig 4B and 4C). Biological processes defined by GO terms common to both HBMECs and L929 cells included vesicle-mediated transport and cellular component organization (Fig 4B and 4C), while membrane organization was associated only with virions purified from HBMECs. STRING protein-protein interaction network analysis revealed an enrichment of cytoskeletal proteins in both cell types (Fig 4D). Myosin-9 (MYH-9) had the highest Sum PEP Score, which provides confidence that the peptide identification is correct, and myosin-9 was associated with virions purified from HBMECs but not L929 cells. Myosin-9 also was identified as a less abundant host protein in lysosome-containing fractions purified from reovirus-infected HBMECs, suggesting that myosin-9 functions in the nonlytic reovirus egress pathway. To further investigate the interaction between myosin-9 and viral particles, we immunoprecipitated myosin-9 from infected HBMECs and probed the immunoprecipitates for reovirus proteins. As shown in S2 Fig, myosin-9 was found to interact with viral outer-capsid protein σ3. These results suggest that myosin-9 physically associates with viral particles in infected HBMECs.

We also purified and analyzed by proteomics the intracellular virions from HBMECs and L929 cells. Most of the top ten candidates associated with the intracellular virion-containing fraction were observed in experiments using both cell lines, including myosin-9 and actin-related proteins (S3A and S3B Fig). The biological processes defined by GO, including intermediate filaments, supramolecular fibers, cytoskeleton organization, and vesicle-mediated transport, are the same for both cell types (S3A and S3B Fig). However, the regulated exocytosis process was associated with intracellular virions purified from HBMECs but not those purified from L929 cells, which could be related to the SO-mediated, nonlytic egress pathway. The STRING protein interaction networks showed functional enrichment of cytoskeleton proteins and similar protein-protein interaction networks in both cell lines (S3C Fig).

### Filaments formed by actin and myosin-9 are associated with virions purified from HBMECs

**Fig 5:**
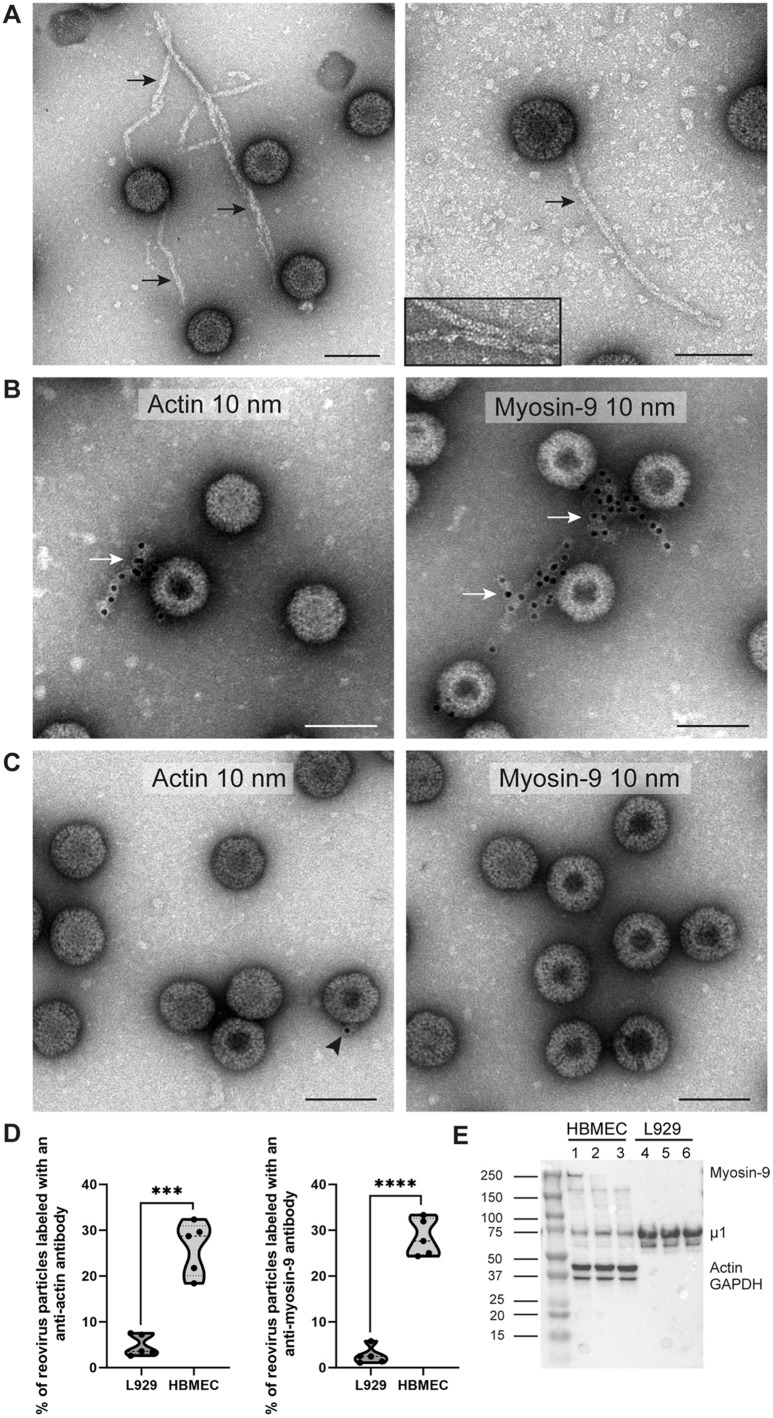
Filaments formed by actin and myosin-9 are attached to reovirus virions purified from HBMECs. HBMECs and L929 cells were infected with reovirus T1LM1-P208S at an MOI of 5 PFU/cell and incubated for 72 h. Intracellular virions were isolated by iodixanol-gradient ultracentrifugation. (A) Purified reovirus virions imaged using TEM are associated with filaments (arrows). The inset shows details of the filament ultrastructure and morphology. Scale bars, 100 nm. (B and C) Purified reovirus virions were immunogold-labeled for actin and myosin-9 using primary antibodies and secondary antibodies conjugated with 10-nm gold particles and imaged using TEM. Filaments marked for actin and myosin-9 (arrows) are associated with virions isolated from HBMECs (B). The majority of reovirus virions purified from L929 cells are not labeled (C). The arrowhead indicates a single gold particle. Scale bars, 100 nm. (D) Percentage of reovirus virions isolated from HBMECs or L929 cells labeled for actin or myosin-9. The number of particles quantified for virions purified from L929 cells, was 437 for actin and 396 for myosin-9. For virions purified from HBMECs, 388 were quantified for actin and 441 were quantified for myosin-9. (E) Immunoblotting of reovirus virions with antibodies specific for myosin-9, reovirus capsid protein µ1, actin, and GAPDH. Reovirus µ1 and GAPDH were used as controls. Lanes 1, 2, and 3, reovirus virions purified from HBMECs. Lanes 4, 5, and 6, reovirus virions purified from L929 cells. Immunoblots are representative of more than three independent experiments.

To determine whether reovirus particles released from infected HBMECs and L929 cells have similar morphology, we used negative staining and TEM. Virions purified from HBMECs were frequently associated with filaments (Fig 5A, black arrows), while those purified from L929 cells were not (S4A and S4B Fig). The structural and proteomics data raise the possibility that the filaments are formed by actin and myosin. To assess this possibility, we used actin and myosin-9 immunolabeling of viral particles purified from HBMECs and L929 cells. The filaments associated with virions purified from HBMECs were labeled with actin and myosin-9 antibodies (Fig 5B, white arrows). These proteins were not detected in association with virions purified from L929 cells (Fig 5C). Quantification of the imaging data showed that a significantly greater number of virion-containing fractions purified from HBMECs were associated with actin- and myosin-9-labeled filaments compared with those purified from L929 cells (Fig 5D). As an immunolabeling control, viral capsid proteins were detected on the surface of viral particles purified from both HBMECs and L929 cells in comparable proportion (S5A and S5B Fig, arrows). Additionally, viral particles disrupted by freezing and thawing after purification from both cell lines had comparable staining for double-stranded RNA to detect the viral genome (S5C Fig, white arrows).

To confirm an association of actin and myosin-9 with virion-containing fractions purified from HBMECs, we used immunoblotting (Fig 5E). Both actin and myosin-9 were detected in virions purified from HBMECs but not L929 cells. However, myosin-9 was not detected in virions purified from HBMECs in all replicates, suggesting that the association of myosin-9 with viral particles is weaker. As controls, the μ1 capsid protein was present in all replicates, while GAPDH was present only in virions purified from HBMECs. These data suggest that reovirus virions released from HBMECs are associated with filaments consisting of actin and myosin-9 and those additional host proteins, such as GAPDH, also associate with released viral particles.

### Electron tomography of reovirus-infected cells reveals mature virions associated with filaments and membranes in reovirus egress compartments

High-resolution 3D imaging of biological samples requires preservation of cellular architecture [29]. To obtain these data, we cryo-immobilized reovirus-infected HBMECs using high-pressure freezing (HPF) and processed the samples by freeze-substitution and embedding in an epoxy resin. Semi-thick sections were imaged using 3D electron tomography. Mature virions in VFs, SOs, and MCs were associated with filaments in infected HBMECs, while immature virions were not, consistent with previous findings [13]. Mature virions were selectively incorporated into SOs, suggesting that the observed filaments function in selection and transport of mature virions to the plasma membrane for nonlytic egress [13]. VFs, SOs, and MCs were distinguished based on morphological features observed by TEM [13]. VFs are identified by their unique electron density, which differs from that of SOs and MCs. They are not enclosed by a defined membrane and contain a mixture of mature and immature viral particles, ER membranes, and microtubules. SOs, in contrast, are modified lysosomes. They are bounded by a membrane and have an electron density that differs from VFs. SOs are enriched in mature virions, which are often associated with filamentous structures, and they contain internal membranes arranged in lamellae and vesicles of varying sizes. These compartments are usually located in close proximity to VFs and establish direct contacts with them [13]. MCs emerge from SOs by budding and are smaller in size. MCs are dispersed in the cytoplasm or near the plasma membrane, where they appear to fuse and release mature virions. MCs contain primarily mature virions associated with filaments and membranes. Thus, the classification of these compartments by electron tomography is supported by differences in electron density, content, size, and subcellular localization.

**Fig 6:**
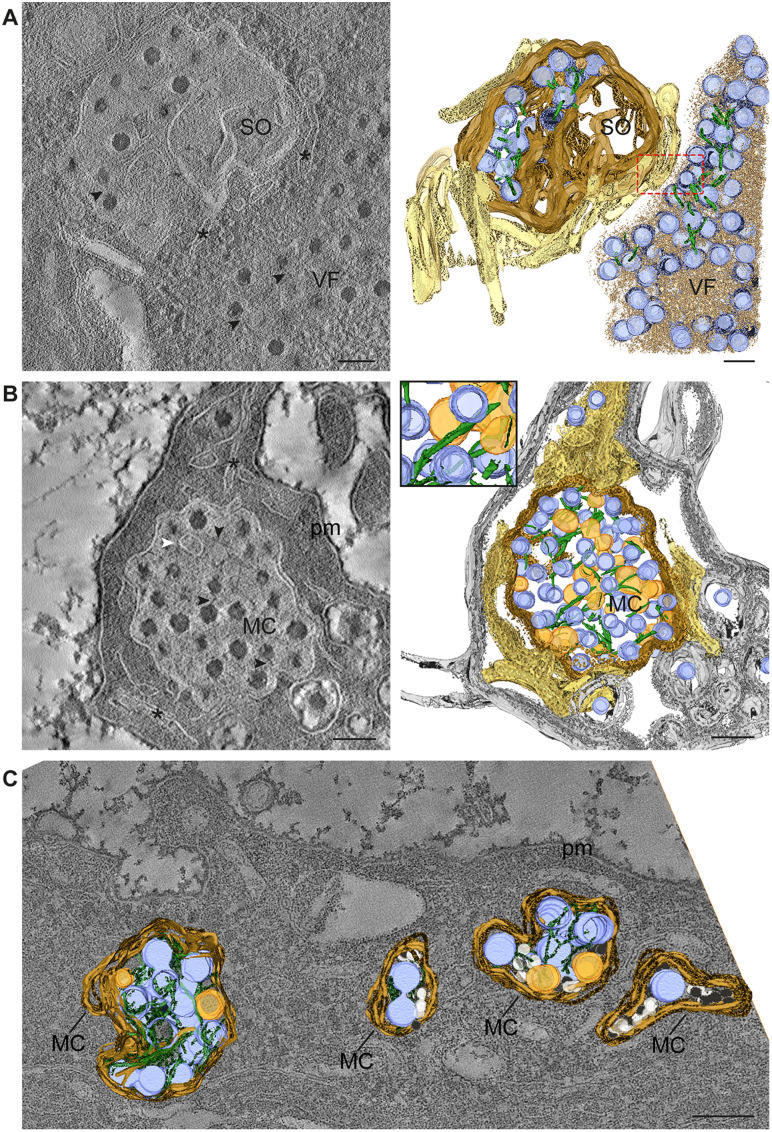
Electron tomography of reovirus-infected cells shows filaments associated with virions inside VFs, SOs, and MCs. HBMECs were infected with reovirus T1L M1-P208S at an MOI of 1 PFU/cell and incubated for 24 h. Cells were processed by high-pressure freezing, freeze-substitution, and semi-thick sectioning. (A and B) Single-tilt scanning transmission electron tomography. (A) Representative tomographic slice and 3D reconstruction of a sorting organelle (SO) adjacent to a viral factory (VF). The SO contains virions and membranes and is surrounded by ER membranes (asterisks) that contact the VF (dashed red box). Virions (blue) inside the VF and SO are associated with filaments (black arrowheads in the tomographic slice or green in the model). (B) Representative tomographic slice and 3D reconstruction of a membranous carrier (MC) adjacent to the plasma membrane (pm). ER membranes (asterisks) surround and connect with the MC. Mature virions are attached to filaments (black arrowheads, image on the left) and a vesicle (white arrowhead). The 3D reconstruction on the right (main field and inset) shows many virions (blue) with filaments (green) attached. (C) Double-tilt transmission electron tomography of four MCs with mature virions (blue) associated with filaments (green) adjacent to the plasma membrane. Vesicles of two different sizes (orange and white) are indicated inside MCs. Color code: SO and MC membranes, brown; ER membranes, yellow; VF membranes, light brown; mature virions, blue; large vesicles, orange; small vesicles, white; pm, grey. Scale bars, 200 nm.

To characterize the 3D-organization of VFs, SOs, and MCs, we used double-tilt TEM tomography and single-tilt scanning transmission electron microscopy (STEM) tomography to improve resolution of the filaments (Fig 6). The tomograms revealed new features of these organelles, including clusters of membranes forming lamellae inside SOs as well as ER membranes surrounding and connecting SOs and MCs (Fig 6A and 6B, respectively, asterisks). The 3D reconstructions showed ER membranes connected to VFs (Fig 6A, dashed red box) and well-defined filaments attached to mature virions and vesicles inside an MC (Fig 6B, black and white arrowheads, respectively). Many virions in SOs and MCs are attached to filaments, suggesting that the filaments are embedded into the virion capsid (Fig 6B, image on the right and inset). We also observed different types of vesicles in MCs (Fig 6C).

### Intracellular distribution and organization of myosin-9 changes with reovirus infection

Our proteomics data identified myosin-9, a cellular protein that functions in vesicular trafficking and cytoskeletal dynamics, as a host factor associated with SOs and virions released from HBMECs. While myosin-9 was not among the most abundant proteins in lysosomes prepared from infected cells, the abundance of myosin-9 associated with lysosomes was altered in a virus-dependent manner. Additionally, myosin-9 was identified as a host protein associated with reovirus particles purified from HBMECs in which reovirus exits using a nonlytic, lysosome-mediated pathway [13], supporting a potential function for myosin-9 in viral egress. We first tested whether reovirus infection induces changes in myosin-9 expression and localization. To visualize myosin-9 distribution, we stained uninfected and reovirus-infected HBMECs using an antibody specific for myosin-9. In uninfected cells, myosin-9 was diffusely distributed in the cytoplasm (S6A Fig). However, in infected cells, myosin-9 redistributed to form long filaments that extend throughout the cell (S6B, S6D, and S6E Fig, asterisks). To visualize the intracellular distribution of myosin-9 and reovirus outer-capsid protein σ3 with higher resolution, we used superresolution stimulated emission depletion (STED) microscopy. In these images, myosin-9 and σ3 have a punctate distribution at the plasma membrane (S6C Fig, white arrowheads). To visualize myosin-9 in real time during infection, HeLa cells were transfected with plasmids encoding σ3-GFP and myosin-9-mCherry, infected with reovirus, and imaged using internal reflection fluorescence (TIRF) microscopy to analyze basal reovirus egress zones (S6F-S6H Fig). Infected cells were identified using phase-contrast microscopy to visualize reovirus factories, which appear as dark, globular structures in a perinuclear region. Myosin-9-mCherry and σ3-GFP had a punctate distribution in the periphery of the cytoplasm at the plasma membrane (S6G and S6H Fig, arrowheads). In uninfected cells analyzed by live-cell imaging, myosin-9-mCherry was diffusely distributed in the cytoplasm (S1 Video), similar to the distribution observed for endogenous myosin-9 in uninfected cells (. S6A Fig). In reovirus-infected cells, identified by phase-contrast microscopy, myosin-9-mCherry reorganizes within the cytoplasm, forming aggregates in discrete areas (S2 Video). The σ3-GFP signal is not shown in the videos, as GFP signal was not detected in the transfected, non-infected cells in this experiment. In infected cells, the green signal could not be captured due to the absence of a control sample required to properly adjust the microscope settings. Thus, this experiment allowed us to compare the myosin-9-mCherry signal in living control and infected cells. Collectively, these data indicate that myosin-9 associates with virion-containing structures during nonlytic egress, suggesting a potential function for myosin-9 in their intracellular transport.

To assess reovirus-induced changes in myosin-9 intracellular distribution with higher resolution, we used TEM to image immunogold-labeled myosin-9 in thawed cryosections (S7 Fig). Myosin-9 was detected in the cytoplasm of uninfected cells and did not appear to associate with specific cellular organelles (S7A Fig, arrowheads). However, in reovirus-infected cells, the myosin-9 signal (S7B Fig, black arrowheads) was detected in VFs and cellular organelles compatible with SOs (S7B Fig, asterisks).

### Reovirus egress is impaired in the absence of myosin-9

**Fig 7:**
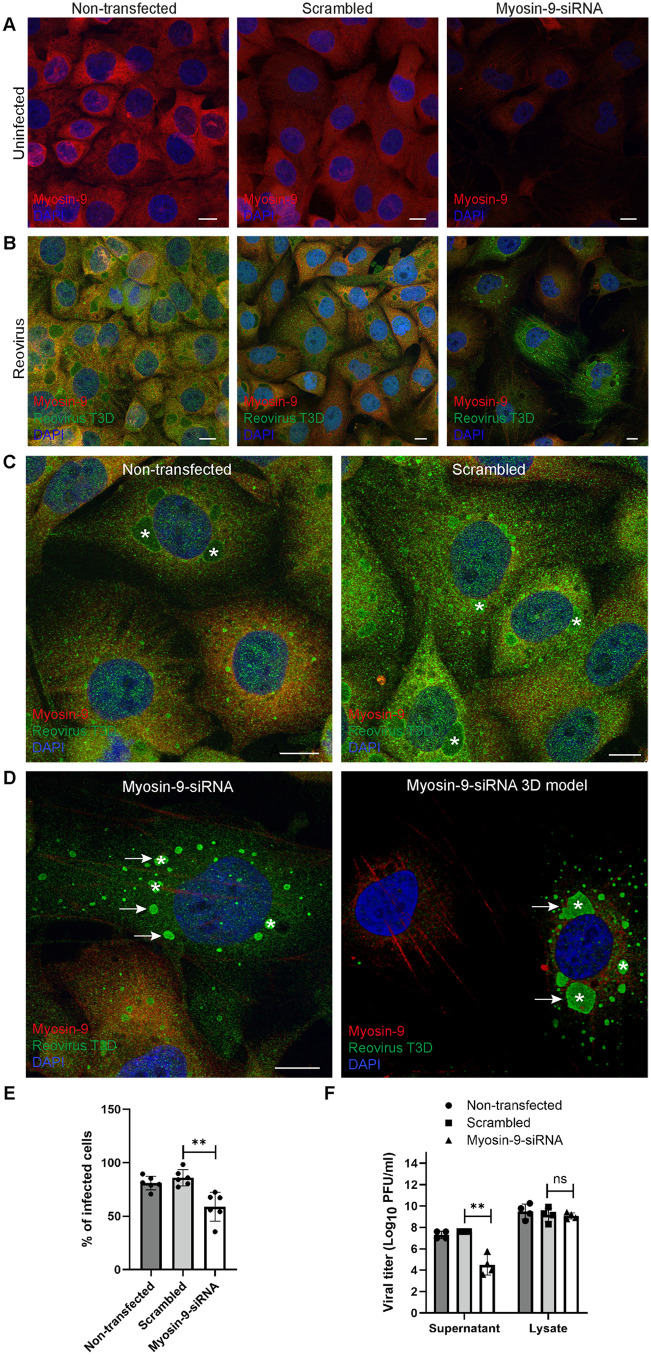
siRNA-mediated knockdown of myosin-9 decreases reovirus infection and egress. HBMECs were non-transfected or transfected with scramble or myosin-9 siRNAs and either uninfected or infected with reovirus T3D at an MOI of 5 PFU/cell at 24 h post-transfection. Cells were incubated for 24 h and imaged using confocal microscopy. (A-D) Immunofluorescence images of the cytoplasmic distribution of myosin-9 and reovirus in mock- and reovirus-infected cells stained with antibodies specific for myosin-9 (red) and reovirus (green). Nuclei were labeled with DAPI (blue). Viral factories are indicated with asterisks. A 3D reconstruction from a series of confocal images is shown in D (image on the right). In cells transfected with myosin-9 siRNA, the fluorescent reovirus signal concentrates primarily inside factories (arrows in D). Myosin-9 redistributes and forms filaments in reovirus-infected cells. Scale bars, 10 µm. (E) Percentage of infected non-transfected and scrambled or myosin-9 siRNA-transfected HBMECs quantified by immunofluorescence staining. (F) Titers of extracellular (supernatant) and intracellular (cell lysate) virus were determined by plaque assay. Data are presented as mean ± SEM for three independent experiments. Significance was determined by unpaired two-tailed student’s *t*-test. ns, not significant; **, *p* < 0.01. Single-channel confocal images are shown in S9 Fig.

To validate a function for myosin-9 in reovirus egress, we used siRNA gene silencing of myosin-9 expression in HBMECs. Although myosins in general play critical roles in cell survival and function [30], we optimized conditions to reduce myosin-9 expression without compromising cell viability or significantly altering other cellular functions (S8 Fig). In cells treated with myosin-9 siRNA, myosin-9 expression was diminished relative to non-transfected cells and cells treated with scrambled siRNA (Fig 7A and 7B). In reovirus-infected cells, myosin-9 and reovirus antigen staining were diffusely distributed in the cytoplasm in non-transfected cells or cells treated with scrambled siRNA (Figs 7C, S9A and S9B). However, in reovirus-infected cells treated with myosin-9 siRNA, there was an accumulation of reovirus antigen in VFs, which was clearly defined in the 3D model (Fig 7D, arrows). Treatment of cells with myosin-9 siRNA significantly reduced the percentage of infected cells and titer of progeny virus released (Fig 7E and 7F). However, myosin-9 siRNA treatment did not alter the titer of intracellular virus. Additionally, relative to non-transfected cells and cells treated with scrambled siRNA, myosin-9 siRNA treatment significantly reduced the expression of reovirus structural and nonstructural proteins (S10A and S10B Fig). Although intracellular viral titers following myosin-9 siRNA treatment were comparable to those in control-treated cells, we observed a marked reduction in the expression of both structural and nonstructural viral proteins. This discrepancy can be explained by the experimental conditions. Cells were infected at a low multiplicity of infection (MOI 5 PFU/cell), resulting in ~ 20% of the cells being infected during the first round at 8 hpi. Under these conditions, efficient viral spread through multiple rounds of infection is required to amplify gene expression and protein production. Additionally, transfected cells are more resistant to infection, further limiting viral propagation. Myosin-9 depletion impaired viral egress, causing virions to accumulate within initially infected cells and preventing their dissemination to neighboring cells. As a result, secondary infections were blocked, reducing the number of newly infected cells and overall viral protein expression. These results are consistent with a function for myosin-9 at post-assembly steps of reovirus replication in the initially infected cells in the culture.

### Silencing of myosin-9 reduces the efficiency of infection with ISVPs

Reovirus enters many types of cells using clathrin-dependent receptor-mediated endocytosis [31]. Myosin-9 can function in endocytosis and maintenance of the actin cytoskeleton [32]. To determine whether myosin-9 functions in reovirus cell entry, we infected cells with infectious subvirion particles (ISVPs), a reovirus disassembly intermediate formed by acid-dependent proteolytic digestion of infecting virions in late endosomes [33,34]. ISVPs also can be prepared *in vitro* by treatment of virions with chymotrypsin [35]. In contrast to virions, ISVPs enter cells at the plasma membrane and bypass the requirement for proteolysis in the endocytic compartment (S11A Fig). Treatment of cells with myosin-9 siRNA but not scrambled siRNA or mock treatment reduced the percentage of infected cells following adsorption with either virions or ISVPs (S11B and S11C Fig). These results suggest that myosin-9 is not required for entry of reovirus into cells, consistent with a role in a post-entry stage of the viral infection cycle.

### Myosin-9 silencing alters lysosome localization

**Fig 8:**
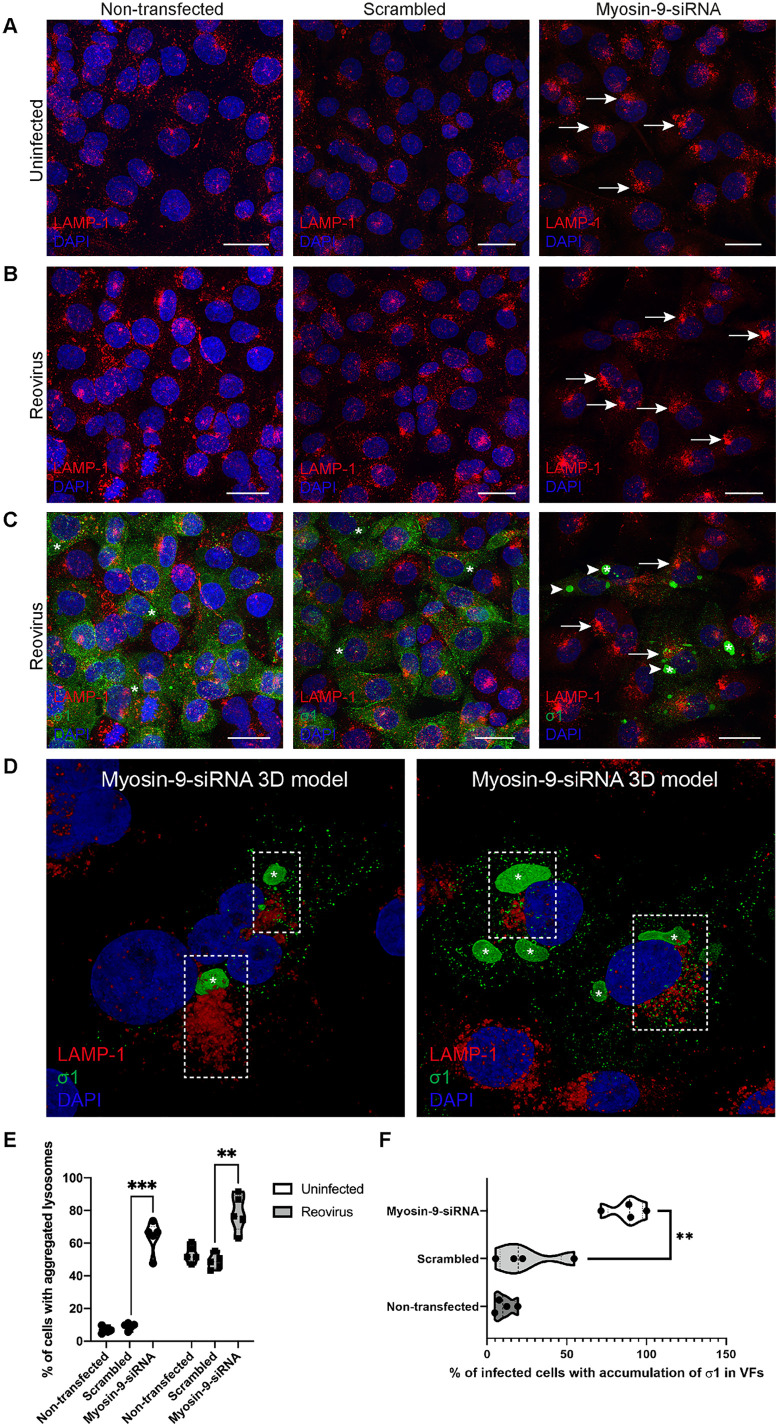
Confocal microscopy of the effect of myosin-9 silencing on the distribution of lysosomes. HBMECs were non-transfected or transfected with scramble or myosin-9 siRNAs and either uninfected or infected with reovirus T3D at an MOI of 5 PFU/cell at 24 h post-transfection. Cells were incubated for 24 h and imaged using confocal microscopy. Immunofluorescence images show cells stained for reovirus capsid protein σ1 (green), LAMP1 (red), and DAPI to label nuclei (blue). (A) In non-transfected or scrambled siRNA-transfected cells, LAMP1 staining (red) was distributed diffusely in the cytoplasm. In myosin-9 siRNA-transfected cells, LAMP1 staining concentrated in the perinuclear region (arrows). (B, C) In reovirus-infected cells, LAMP1 staining concentrated in specific areas adjacent to the nucleus (arrows). (C) Reovirus σ1 staining distributed in the cytoplasm in non-transfected or scrambled siRNA-transfected cells and inside reovirus factories (asterisks) in cells in which myosin-9 expression was silenced (arrowheads). Scale bars, 25 µm. (D) 3D reconstructions from a series of confocal images. Dashed boxes indicate areas in which LAMP1 and σ1 concentrate in the frontal projection of the volume. (E and F) Quantification of cells with aggregated lysosomes in non-transfected, scrambled-siRNA-transfected, and myosin-9-siRNA-transfected cells uninfected or infected with reovirus. Cells with LAMP1 protein concentrated in a specific area of the perinuclear region and cells with σ1 mostly distributed inside reovirus factories rather than diffusely in the cytoplasm were enumerated. The results are presented as the percentage of cells with those distributions of fluorescent signals ± SEM for three independent experiments. Significance was determined by unpaired two-tailed student’s *t*-test. **, *p* < 0.01 and ***, *p* < 0.001. High-magnification confocal images of panels A and B are shown in S12Fig.

During reovirus infection, lysosomes are modified and redistribute to the periphery of VFs [13]. To determine whether myosin-9 is required for lysosome redistribution, we stained infected cells with an antibody specific for lysosomal marker LAMP-1 and reovirus protein σ1 and imaged cells using confocal microscopy. In uninfected cells that were untreated or treated with scrambled siRNA, LAMP-1-positive organelles were diffuse in the cytoplasm (Fig 8A left and middle panel). In contrast, in cells treated with myosin-9 siRNA, LAMP-1-positive organelles were distributed in a perinuclear region (Figs 8A right panel, S12A). Reovirus-infected cells show a comparable phenotype, characterized by perinuclear accumulation of lysosomes in myosin-9 siRNA-treated cells relative to untreated controls. (Fig 8B). In reovirus-infected cells that were untreated or treated with scrambled siRNA, σ1 was distributed in the cytoplasm (Fig 8C left and middle panel). However, σ1 accumulated in VFs (asterisks) in cells treated with myosin-9 siRNA (Figs 8C right panel, arrowheads, and S12B). VFs are readily distinguishable in the cytoplasm of infected cells by phase-contrast microscopy as globular structures with a perinuclear distribution. Based on their morphology, position, and size, we conclude that σ1 accumulates in VFs in myosin-9 siRNA-treated cells. The distribution of lysosomes in a perinuclear region and accumulation of σ1 in infected cells treated with myosin-9 siRNA is shown in the 3D models obtained from individual confocal micrographs (Fig 8D, dashed boxes). The percentage of cells with aggregated lysosomes significantly increased in both uninfected and infected cells treated with myosin-9 siRNA (Fig 8E). The number of cells with σ1 concentrated in VFs also increased significantly following treatment with myosin-9 siRNA (Fig 8F). These data suggest that lysosome localization and σ1 distribution in HBMECs requires myosin-9.

### Myosin-9 maintains VF structure and SO function during infection

**Fig 9:**
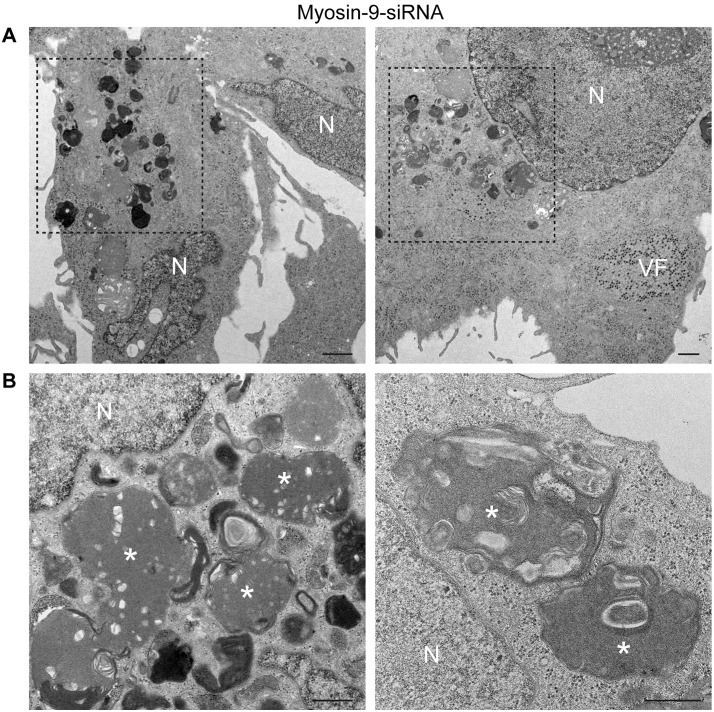
Electron microscopy of the effect of myosin-9 silencing on the distribution of lysosomes in reovirus-infected cells. HBMECs were transfected with myosin-9 siRNAs and infected with reovirus T3D at an MOI of 5 PFU/cell at 24 h post-transfection. Cells were incubated for 24 h and processed for TEM. (A) Lysosomes are concentrated near the nucleus (dashed boxes), and a VF was observed in the same plane as aggregated lysosomes. Scale bars, 1 µm. (B) Higher-magnification images of aggregated lysosomes (asterisks). N, nucleus. Scale bars, 500 nm.

**Fig 10:**
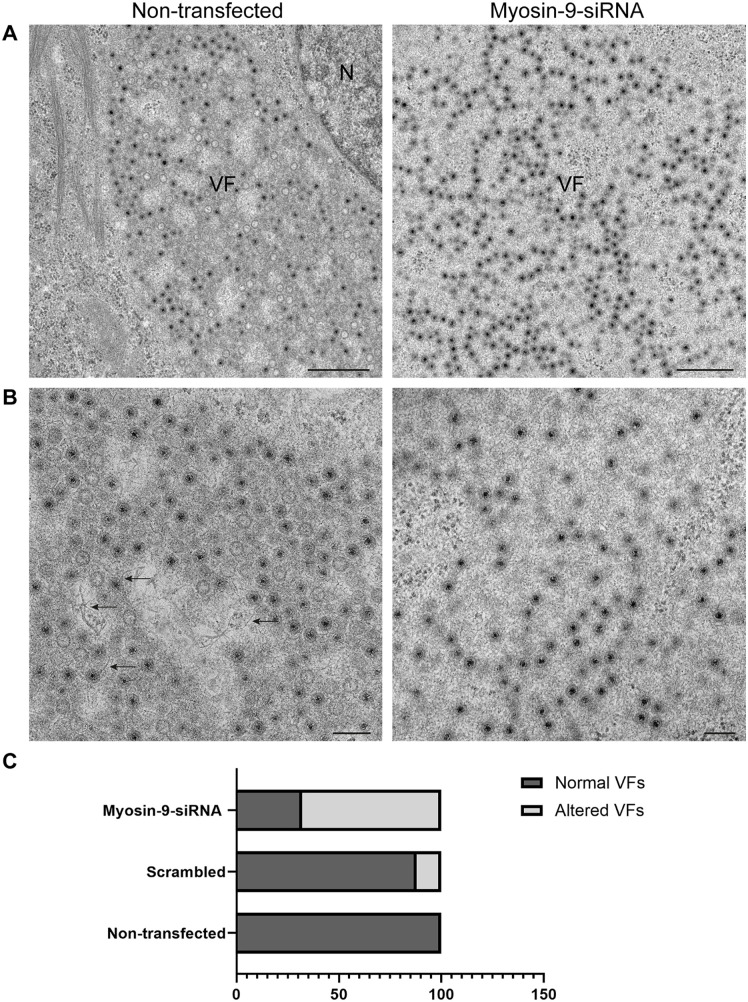
Electron microscopy of the effects of myosin-9 silencing on the morphology of reovirus factories. HBMECs were non-transfected or transfected with scramble or myosin-9 siRNAs and either uninfected or infected with reovirus T3D at an MOI of 5 PFU/cell at 24 h post-transfection. Cells were incubated for 24 h and processed for TEM. (A) VFs in infected cells. N, nucleus. Scale bars, 500 nm. (B) Higher-magnification images of VFs. VFs in non-transfected cells contained electron-dense material compacting mature and immature virions. Arrows point to filaments associated with mature virions. VFs in cells transfected with myosin-9 siRNAs did not contain electron-dense material compacting viral particles, and no filaments were detected. Scale bars, 200 nm. (C) Quantification of normal and altered VFs observed in non-transfected cells or cells transfected with scramble or myosin-9 siRNAs. A total of 50 cells per condition was counted.

To determine how myosin-9 inhibition affects cellular architecture, HBMECs were treated with myosin-9 siRNA, scrambled siRNA, or untreated and mock-infected or infected with reovirus. Cells were fixed and imaged by TEM. Lysosomes were randomly distributed in the cytoplasm of mock-infected, untreated cells, or cells treated with scrambled siRNA (S13A-S13F Fig, asterisks). Higher-magnification images showed the typical morphology of lysosomes with internal electron-dense material (S13C and S13F Fig). In contrast, lysosomes were distributed near the nucleus in cells treated with myosin-9 siRNA (S13G Fig, dashed box). However, lysosome morphology and internal electron-dense contents were not affected by myosin-9 knockdown (S13H and S13I Fig). In reovirus-infected cells not treated with siRNA, lysosomes were adjacent to VFs positioned to collect mature virions (S14A-S14D Fig). However, reduction of myosin-9 expression in infected cells was associated with the accumulation of lysosomes near the nucleus (Fig 9A, dashed boxes). Serial sections were analyzed to localize the planes in which lysosomes accumulate. Using this approach, we observed that large groups of lysosomes did not distribute in the same plane or adjacent to VFs. In higher-magnification images, lysosomes (asterisks) showed the characteristic electron-dense internal contents and membranes, but neither virions nor MCs were observed (Fig 9B). TEM images revealed that VFs in cells in which myosin-9 was silenced were dismantled and contained less electron-dense material relative to control cells (Fig 10A). Higher-magnification images of VFs in non-transfected cells show numerous filaments attached to mature virions (Fig 10B, arrows in image on the left). However, no filaments were observed inside VFs in cells transfected with myosin-9-siRNA (Fig 10B, image on the right). Quantification of these findings demonstrated that myosin-9 gene silencing increased the number of altered VFs (Fig 10C).

As a complementary approach to define the function of myosin-9 in reovirus replication, we used blebbistatin, a selective cell-permeable myosin-9 ATPase inhibitor. Uninfected or reovirus-infected HBMECs were incubated at 17 h post-adsorption with or without blebbistatin for 1 h. Cells were stained with antibodies specific for reovirus σ3 and LAMP-1 and imaged using confocal and electron microscopy (S15 Fig). In untreated cells infected with reovirus, σ3 staining was diffuse throughout the cytoplasm, and lysosomes were localized at the periphery of VFs (S15A Fig). However, in blebbistatin-treated cells, σ3 was primarily located in VFs, and LAMP1 staining was weak (S15A Fig, asterisks). Using TEM, we observed that blebbistatin treatment affected SO and MC formation, and no mature virions were detected inside these egress organelles (S15B and S15C Fig). Higher-magnification views revealed that SOs and MCs in untreated cells contained mature virions, while SOs and MCs in blebbistatin-treated cells were filled with empty capsids and immature viral particles (S15D Fig). Collectively, these data indicate that myosin-9 is not required to maintain lysosome morphology but is required for lysosome distribution and incorporation of mature virions in SOs. VFs also are altered and contain less electron-dense material when myosin-9 expression is reduced. However, inhibition of myosin-9 ATPase activity by blebbistatin impairs formation of SOs and MCs, and these egress organelles fill with immature virions. Thus, myosin-9 is required for sorting mature virions from VFs into SOs.

## Discussion

During reovirus infection, modified lysosomes or SOs are recruited to VFs, a process that may be mediated by cytoskeleton proteins. In this study, we purified lysosomes from uninfected and reovirus-infected cells to identify host proteins associated with lysosome-mediated, nonlytic viral egress. We found that proteins less abundant in lysosome-containing fractions from infected cells are associated with the cytoskeleton, including myosin-9. Myosin-9 also was identified as a protein associated with virions released using the lysosome-mediated, nonlytic egress pathway. By diminishing myosin-9 expression using siRNA, we demonstrated that myosin-9 functions in selective transport of reovirus virions through the intracellular lysosome-mediated, nonlytic egress pathway.

Myosins constitute a large superfamily of molecular motor proteins that bind actin and generate force and movement by ATP hydrolysis. Myosin-9 belongs to the myosin-2 subfamily, which includes muscle and non-muscle myosins [36]. In non-muscle cells, myosin-9 associates with actin filaments to form distinct actomyosin structures that function in cellular processes that rely on rapid actomyosin remodeling, including maintenance of the cytoskeleton, cell migration, adhesion, and motility [37–41].

Myosin-9 is required for cell entry of several enveloped viruses, including Epstein-Barr virus (EBV) [42], HSV-1 [37], influenza A virus [43], porcine reproductive and respiratory syndrome virus [44], SARS-CoV-2 [45], and severe fever with thrombocytopenia syndrome virus [46]. During EBV and HSV-1 infection, myosin-9 redistributes from the cytoplasm to the plasma membrane where it serves as a receptor or co-receptor [37,42,47]. In our study, we found that myosin-9 localizes to long filaments and not the plasma membrane during reovirus infection. These filaments are in close proximity to VFs and span the cytoplasm (7D and S6B, S6D and S6E Figs), suggesting that they function in reovirus transit during entry or egress. Myosin-9 functions in intracellular vesicular trafficking and is required for efficient transport of incoming HSV-1 nucleocapsids to the nucleus [48]. Our data suggest that myosin-9 is not required for reovirus entry. Diminished myosin-9 expression significantly reduced reovirus infection following adsorption with either virions or infectious subvirion particles (ISVPs) (S11 Fig), which bypass a requirement for clathrin-dependent uptake into endosomes [31]. However, diminished myosin-9 expression significantly reduced the titers of virus released into the culture supernatant, while titers of virus in cell lysates were not altered (Fig 7F). We also observed a significant reduction in expression of viral nonstructural and structural proteins in cells treated with myosin-9 siRNA (S10 Fig). The discrepancy between expression of reovirus proteins and intracellular viral titer supports a function for myosin-9 in egress. Reovirus can replicate and assemble in cells with reduced myosin-9 expression. However, virus egress is impaired, which limits dissemination and amplification of infection and is reflected in reduced levels of viral protein expression. These data are consistent with a function for myosin-9 in nonlytic reovirus egress.

The reovirus nonlytic egress pathway in HBMECs is comprised of membranous organelles referred to as SOs and MCs [13]. SOs are modified lysosomes recruited to the periphery of VFs during infection. Mature virions are selectively shuttled from VFs to SOs for transit to the cell surface in MCs. Immunogold labeling showed myosin-9 in VFs as well as in cellular compartments compatible with SOs and MCs (S7 Fig), consistent with a function for myosin-9 in nonlytic egress. Lysosomal transport and distribution are regulated by several mechanisms, including the actin cytoskeleton and myosin motor proteins [49]. Diminished myosin-9 expression led to aggregation of lysosomes near the nucleus in both uninfected and infected cells (Figs 8 and 9A). This phenotype resembles that of non-transfected cells infected with reovirus [13]. These findings suggest that myosin-9 functions in regulating lysosome distribution during viral infection, potentially facilitating recruitment of these organelles to viral replication sites. The similarity in lysosome distribution following myosin-9 knockdown and reovirus infection raises the possibility that the virus exploits host cytoskeletal machinery, including myosin-9, to redirect lysosomal trafficking. Furthermore, SOs did not distribute to VFs and were often not in the same plane as VFs in infected cells with diminished myosin-9 expression, providing additional evidence that myosin-9 functions in lysosome transport and is required for distribution of SOs to VFs. Disruption of SO distribution to the periphery of VFs led to a significant increase in reovirus protein staining intensity in VFs relative to control cells (Figs 7D and 8B–8D). These results demonstrate that altering the transport of lysosomes to VFs prevents sorting of mature virions from VFs to SOs and subsequent transit through the nonlytic egress pathway and instead leads to accumulation of mature virions in VFs.

Mature virions in VFs, SOs, and MCs in HBMECs are associated with filaments. These filaments only associate with extracellular virions purified from cells that release reovirus using the lysosome-mediated, nonlytic egress pathway, suggesting that the filaments function in selective sorting and release of mature virions using this egress mechanism. We found that the filaments are composed of actin and myosin-9 (Fig 5B), suggesting that myosin-9 regulates the nonlytic reovirus egress pathway in HBMECs. In cells with reduced myosin-9 expression, the integrity of VFs was lost, and filaments attached to virions were not observed (Fig 10). Similar observations were made using cells treated with blebbistatin, which inhibits myosin-9 ATPase activity. However, in blebbistatin-treated cells, SOs were recruited to VFs, but only empty particles were detected inside SOs and MCs (S15 Fig). These data suggest that myosin-9 has at least two functions in reovirus nonlytic egress, transport of SOs to VFs and selection of mature virions for release. Furthermore, these data suggest that myosin-9 ATPase activity is required to facilitate transport of mature reovirus virions from VFs to SOs. Other viruses, including the parvovirus, minute virus of mice [50], and SARS-CoV-2 [51], also use egress pathways that rely on modified lysosomes. Minute virus of mice additionally depends on the cytoskeleton or actin-related proteins to regulate egress [50]. These data suggest that the reovirus egress pathway studied here may be a common mechanism of viral egress.

High-resolution electron tomograms of VFs, SOs, and MCs revealed ultrastructural elements not previously observed. Using a combination of TEM and STEM tomography, we observed filaments associated with mature virions in VFs, SOs, and MCs. Additional high-resolution studies are required to identify macromolecular complexes associated with virions and filaments that localize in the VF-SO contact sites. We also visualized ER membranes surrounding SOs and MCs. These ER membranes could mediate contacts between VFs and SOs, facilitating transport of mature virions into SOs. In support of ER membranes forming contact sites with VFs, ER-associated proteins were enriched in lysosomal fractions purified from reovirus-infected cells (Fig 3), including kinectin 1 (KTN1). KTN1 functions in ER distribution and communication between the ER and lysosomes in neurons [52], suggesting that this protein could mediate contacts between ER, VFs, and SOs during reovirus infection. Other proteins identified in our screens are required for protein folding and other ER functions. Future studies are necessary to determine whether these proteins also are required for formation of the membrane contacts between the ER and SOs observed in reovirus-infected cells. In addition, vesicles of varying sizes were observed inside SOs and MCs. Several nonenveloped viruses exit cells using an MVB-exosomal pathway, including norovirus [3] and HEV [6]. However, the function of these vesicles within reovirus egress compartments appears to be distinct from that of exosomes in the MVB pathway. The vesicles present inside SOs and MCs are located near virions, but they do not contain virions or appear to transport them. Further research is required to determine functions of these vesicles in reovirus nonlytic egress.

Findings made in this study demonstrate an essential role for myosin-9 in facilitating lysosome-mediated, nonlytic reovirus egress. Mysoin-9 is required for recruitment of lysosomes to VFs and collection of mature virions into the nonlytic egress machinery. The filaments attached to mature virions are formed by actin and myosin-9 and accompany mature virions in every egress compartment. Engagement of virions in the nonlytic egress pathway by actin/myosin-9 filaments, together with blockade of virus egress by myosin-9 gene silencing, suggests that the filaments have a structural and selection role in nonlytic viral egress. The identification of myosin-9 in virion-containing fractions and its interaction with the viral protein σ3, as demonstrated by co-immunoprecipitation, provides evidence for a functional association between this cytoskeletal motor protein and reovirus particles. Given that myosin-9 participates in intracellular trafficking and vesicle transport, its interaction with σ3 suggests a role in guiding virions through the nonlytic egress pathway in HBMECs. This model is consistent with previous observations of filament-associated virions within viral factories and egress compartments. The absence of myosin-9 association in L929 cells, which predominantly undergo lytic egress, further supports the cell-type-specific involvement of myosin-9 in nonlytic viral release. These findings expand an understanding of host cytoskeletal components in reovirus infection and highlight myosin-9 as a candidate mediator of virus transport and secretion in endothelial cells. One limitation of our study is the inability to perform complementation experiments using an siRNA-resistant myosin-9 construct. Despite repeated attempts, we were unsuccessful in engineering a myosin-9 cDNA variant that could evade siRNA-mediated knockdown. This technical challenge prevented us from directly validating the specificity of the observed phenotypes.

Important questions remain. How are mature virions selectively recognized by actin/myosin-9 filaments and shuttled into SOs? How do MCs bud from SOs, transport to the cell surface, and fuse with the plasma membrane? What functions, if any, do the filaments have following viral release? Are actin and GAPDH involved in nonlytic reovirus egress? Our ongoing work seeks to answer these questions and determine how the lysosome-mediated egress pathway contributes to reovirus pathogenesis.

## Materials and methods

### Cells and viruses

HBMECs (obtained from Kwang Sik Kim, Johns Hopkins University) [53] were maintained in RPMI medium 1640 supplemented to contain 10% FBS (BI Biological Industries), 10% Nu Serum (Gibco), 1% MEM nonessential amino acids (Sigma-Aldrich), 1% L-glutamine (Merck), 1% penicillin/streptomycin (Sigma-Aldrich), and 0.1% amphotericin B (Sigma-Aldrich). Spinner-adapted murine L929 fibroblasts (ATCC; CCL-1) were maintained in Dulbecco’s modified Eagle medium (DMEM) (Sigma-Aldrich; D6429) supplemented to contain 10% FBS, 1% MEM nonessential amino acids (Sigma-Aldrich), 1% L-glutamine (Merck), and 1% penicillin/streptomycin (Sigma-Aldrich). HeLa cells (ATCC; CCL-2) were maintained in DMEM (Sigma-Aldrich; D6429) supplemented to contain 10% FBS, 1% sodium pyruvate (Gibco), 1% MEM nonessential amino acids (Sigma-Aldrich), 1% L-glutamine (Merck), 1% penicillin/streptomycin (Sigma-Aldrich), and 0.1% amphotericin B (Sigma-Aldrich).

Cells were infected with reovirus strain T3D or T1L M1-P208S, which is identical to the prototype T1L strain except for a proline-to-serine substitution at position 208 of the μ2 protein (M1 gene). This substitution changes factory morphology from filamentous to globular [54], making factories more apparent for imaging experiments. For some experiments (Figs 7, 8, 9, 10, S1, S2, S7, S8, S9, S10, S12 and S14 Figs), due to the availability of specific antibodies, cells were infected with reovirus strain T3D. This virus was recovered by plasmid-based reverse genetics [55]. Both T3D and T1L M1-P208S produce globular factories and have similar infection dynamics, allowing complementary analyses using either virus strain. Reovirus strains were purified by cesium gradient centrifugation as described previously [56] and propagated at an MOI of 5 PFU/cell at 33°C for 65 h to generate working stocks. Viral titers were determined by plaque assay using L929 cells [57]. ISVPs were prepared by treatment of 2 x 10^12^ purified T1L M1-P208S reovirus particles with chymotrypsin (Sigma-Aldrich; C3142) diluted to 200 μg/mL at 37°C for 60 min. The digestion was terminated by addition of PMSF to a final concentration of 2 mM [35]. Virion-to-ISVP conversion was confirmed by SDS-PAGE and colloidal blue staining to assess the loss of σ3 and cleavage of μ1C to δ.

### Infections and blebbistatin treatment

Cells were adsorbed with reovirus at MOIs of 1 or 5 PFU/cell (diluted in culture medium) at 37°C for 1 h. Inoculum was removed, and cells were incubated at 37°C for various times post adsorption in fresh medium supplemented to contain 2% FBS.

HBMECs were either mock infected or adsorbed with T1L M1-P208S at an MOI of 1 PFU/cell, treated at 17 h post adsorption with 50 μM blebbistatin (Sigma-Aldrich; B0560), and incubated for 1 h. Cells were fixed and processed for confocal and electron microscopy.

### siRNA and DNA transfections

HBMECs were reverse-transfected with myosin-9 siRNA duplexes (Human, Locus ID 4627, Origene; SR303039) or the Trilencer-27 universal scrambled siRNA duplex (Origene; SR30004) at a final concentration of 2 pM each using Lipofectamine RNAiMax (Thermo Fisher Scientific; 13778075). 24 h post-transfection, cells were either mock-infected or adsorbed with reovirus strain T3D, due to the availability of specific antibodies for fluorescence microscopy, at an MOI of 5 PFU/cell and incubated for 24 h. Cells were analyzed by western blot and confocal and electron microscopy. Intracellular and extracellular viral titers were determined by plaque assay using L929 cells. Non-transfected cells were processed and used as control.

HeLa cells cultivated on glass-bottom culture p35 plates (Ibidi) were transfected with plasmids encoding T3D σ3-GFP [13] or pCMV-mCherry-MHC-IIA (gift from Venkaiah Betapudi [58], Addgene; #35687) using FuGene 6 (Promega, E2691) at a 3:1 FuGene 6: DNA ratio. At 24 h post-transfection, cell monolayers were adsorbed with reovirus T1L M1-P208S at an MOI of 5 PFU/cell for 24 h, and imaged using total TIRF microscopy.

### Isolation of lysosomes using iodixanol

HBMECs were seeded into P150 plates to obtain a confluency of 100% in three days. Cells were either mock-infected or adsorbed with reovirus strain T1LM1-P208S at an MOI of 1 PFU/cell and incubated at 37 °C for 48 h. Cell monolayers were washed with 5 ml of cold phosphate buffered saline (PBS) and harvested in 5 ml of PBS using a cell scraper. Cells were centrifuged at 1,400 rpm at 4°C for 5 min. Pelleted cells were suspended in homogenization buffer (0.25 M sucrose, 1 mM EDTA, 20 mM HEPES-NaOH, pH 7.4, and protease inhibitors (Roche, 11836170001)) and homogenized on ice using 15 gentle strokes of the pestle of a loose-fitting Dounce homogenizer. Homogenization was monitored with a light microscope. For preparation of the light mitochondrial fraction, the homogenized sample was centrifuged at 1,000 x *g* at 4°C for 10 min to pellet nuclei, cell debris, and unbroken cells. The supernatant was collected and stored on ice. The pellet was resuspended in 3 ml of homogenization buffer, homogenized using 2–3 gentle strokes of the pestle of a loose-fitting Dounce homogenizer and centrifuged at 1,000 x *g* at 4°C for 10 min. The two 1,000 x *g* supernatants were combined and centrifuged at 3,000 x *g* at 4°C for 10 min to pellet the heavy mitochondria. The supernatant was collected and kept on ice. The pellet was resuspended in homogenization buffer and centrifuged at 3,000 x *g* at 4°C for 10 min. The two 3,000 x *g* supernatants were combined and centrifuged at 20,000 x *g* at 4°C for 10 min to pellet the light mitochondria. The light mitochondrial pellet was resuspended in 1 ml of homogenization buffer and centrifuged at 20,000 x *g* at 4°C for 10 min to pellet lysosomes. The pellet containing the lysosomal fraction, was suspended in 1 ml of homogenization buffer and used for the gradient input.

A discontinuous gradient of 4, 8, 10, 16, 24% (w/v) iodixanol (OptiPrep) diluted in homogenization buffer was prepared and stabilized at room temperature (RT) for 1 h. The sample was loaded at the top of the gradient and centrifuged at 145,000 x *g* at 4°C for 2 h. Lysosomes banded close to the top of the gradient and were collected with a needle and syringe and resuspended in 5 ml of homogenization buffer. The resuspended lysosomal fraction was centrifuged at 32,500 x *g* at 4°C for 30 min and resuspended in 200–300 µl of homogenization buffer to concentrate the sample. Fractions were analyzed by negative staining and immunoblotting with the lysosomal marker LAMP1. Lysosomes were stored at -80ºC for proteomic analysis or 4ºC for electron microscopy studies.

### Purification of extracellular and intracellular reovirus using iodixanol

Forty p150 plates of HBMECs or L929 cells were maintained in 25 mL RPMI or DMEM supplemented to contain 10% FBS, respectively. When cells reached 80% confluency, they were infected with reovirus TL1M1-P208S at an MOI of 5 PFU/cell at 37ºC for 48 or 72 h to purify the intracellular or extracellular virions respectively. The culture medium containing extracellular virions was clarified by centrifugation at 4,000 x *g* at 4ºC for 20 min and stored at 4ºC until cell monolayers were processed. Cell monolayers were washed with 5 ml of homogenization medium (250 mM NaCl, 9 mM β-mercaptoethanol, 10 mM Tris-HCl, pH 7.4, and protease inhibitors) and collected into 50 ml conical tubes using a cell scraper and 5 ml of fresh homogenization medium. To collect intracellular virions, the cells were freeze/thawed three times followed by sonication (3 x 20 sec pulses using a bath sonicator). The samples were clarified by centrifugation at 4,000 x *g* at 4ºC for 20 min and supernatants were passed through a 0.2 µm filter to eliminate cell debris and cell genome.

To concentrate virions prior to gradient purification, the supernatants containing extracellular or intracellular virions were centrifuged through a 30% (w/v) sucrose cushion (sucrose diluted in NaCl solution (0.85% NaCl, 10 mM Tris-HCl, pH 7.4)) at 80,000 x *g* at 4°C for 2 h. Virus pellets were resuspended in NaCl solution supplemented to contain protease inhibitors. The number and structure of viral particles in the concentrated samples were monitored by negative staining and electron microscopy.

A discontinuous gradient was prepared using 50% (w/v) iodixanol (OptiPrep) diluted in NaCl solution supplemented to contain protease inhibitors. The gradient was made with two fractions of 2 ml of 40 and 41% iodixanol and stabilized for 1 h at RT. The samples were loaded on the gradient and centrifuged at 107,170 x *g* at 4°C overnight. The virus band was collected using a needle and a syringe, confirmed by negative staining, and stored at -80ºC. Reovirus virions purified from different cell lines were analyzed by immunoblotting, proteomics, and electron microscopy.

### Proteomics

To identify cellular factors associated with reovirus egress, purified lysosomes and reovirus virions were analyzed by proteomics. The digestion of the samples was performed using trypsin and S-Trap columns. Each digested sample was cleaned using a StageTip C18. Peptides were separated by polarity using liquid chromatography for 60 min, and fragmented with the mass spectrometer Orbitrap Exploris 240 (LC-MS/MS). The “raw data” were exported and analyzed using the software MASCOT and the *Homo sapiens* and *Orthoreovirus T1L* proteomes from Uniprot. Selected proteins had a q-value <0.01 and at least two PSMs or peptides identified. Analysis of purified lysosomes included normalized abundance as a quantification and comparison parameter. Normalized abundance was calculated as the sum of the abundance (or intensities) of all peptides assigned to each protein. Potential candidates were chosen from lists of proteins with higher Sum PEP Score values that fulfil the previous conditions (q-value <0.01 and at least two PSMs or peptides identified). Identified genes were analyzed using Gene Ontology (GO) and STRING (functional protein association networks) tools in order to show biological processes and protein interactions, respectively.

### Immunoblotting

Cells were harvested using CellStripper reagent (Corning; 25–056-CI) and lysed with RIPA lysis buffer (Thermo Fisher Scientific; 89900) supplemented to contain protease inhibitors. Total protein in cell lysates, purified lysosomes, and purified virions was quantified using Pierce BCA Protein Assay Kit (Thermo Fisher Scientific; 23227) according to the manufacturer’s specifications. Cell lysates (5 or 10 µg) were mixed with 4X Laemmli sample buffer supplemented to contain 5% β-mercaptoethanol and incubated at 95°C for 10 min. Proteins were resolved by SDS-PAGE and transferred to polyvinylidene fluoride (PVDF) membranes by standard blotting procedures. Membranes were blocked using 5% non‐fat dry milk in PBS supplemented to contain 0.05% Tween 20 (blocking buffer) at 4°C overnight and incubated at RT for 1 h with primary antibody diluted in blocking buffer. The following primary antibodies were used at optimized concentrations for immunoblotting: rabbit anti-LAMP-1 polyclonal antibody (1:1,000) (Abcam; ab24170), mouse anti-non-muscle Myosin IIA monoclonal antibody [2B3] (1:1,000) (Abcam; ab55456), mouse anti-β-actin monoclonal antibody (1:2,000) (Sigma-Aldrich; A5441), mouse anti-GAPDH monoclonal antibody (1:2,000) (Abcam; ab8245), mouse 8H6 anti-μ1 monoclonal antibody (1:1,000), rabbit anti-reovirus polyclonal antiserum specific for T3D (1:1,000), rabbit VU82 anti-σNS polyclonal antibody (1:1,000), rabbit anti-tubulin polyclonal antibody (1:1000) (Sigma-Aldrich; T3526), and chicken anti-μNS polyclonal antibody (1:10,000) (provided by John Parker [59]; Cornell University). Membranes were washed with PBS supplemented to contain 0.05% Tween 20 and incubated with species-specific secondary antibodies conjugated with horseradish peroxidase (HRP) at RT for 1 h. Immunoblots were visualized using ECL solutions (SuperSignal West Dura Extended Duration Substrate, Thermo Fisher Scientific; 34076) with a ChemiDoc Imager (BioRad). Band intensity of proteins was calculated using Image Lab software (BioRad) and normalized to loading controls. Three independent replicas were analyzed in each experiment.

### Co-immunoprecipitation

HBMEC cells were adsorbed with purified reovirus at MOI of 500 PFU/cell or PBS (diluted in culture medium) at 37°C for 1 h. Inoculum was removed, and cells were incubated at 37°C for 24 h post adsorption in fresh medium supplemented to contain 2% FBS. Cells were lysed with Co-IP lysis buffer (20 mM Tris-HCl, 137 mM NaCl, 0.1% NP40, 2 mM EDTA) at 4°C for 1 h using an end-to-end rotator. Cell lysates were passed through a 25-gauge needle ten times, and debris was collected by centrifugation at 17,000 x *g* for 10 min. Clarified lysates were incubated with 1 μg mouse anti-non-muscle Myosin IIA monoclonal antibody [2B3] (Abcam; ab55456) or 1 μg rabbit IgG isotype antibody (Invitrogen, #02–6102) at 4°C overnight using an end-to-end rotator followed by incubation with Protein G Dynabeads (Thermo, #10004D) at 4°C for 1 h.

Proteins were resolved by SDS-PAGE using Mini-Protean TGX precast gels (Bio-Rad) and transferred to PVDF membranes (Bio-Rad, #1704156) using a Trans-Blot Turbo Transfer System (Bio-Rad) according to the manufacturer’s instructions. Membranes were blocked using 5% BSA in tris-buffered saline (TBS) at room temperature for 1 h and incubated overnight with mouse 4F2 anti σ3 monoclonal antibody (1:2,000) diluted in TBS supplemented to contain 0.1% Tween 20 (Sigma-Aldrich, #P7949) (TBS-T) at 4°C. Membranes were washed three times with TBS-T and incubated with species-specific secondary antibodies conjugated with IRDye800CW or IRDye680LT (LI-COR) diluted 1:10,000 in TBS-T. Immunoblots were visualized using an Odyssey CLx imager (LI-COR).

### Immunofluorescence, confocal, STED, and TIRF microscopy

For confocal and STED experiments, cells were grown on sterile glass coverslips, fixed with 4% PFA (TAAB Laboratories), and washed three times with cytoskeleton buffer (150 mM NaCl, 10 mM MES, 5 mM MgCl, 5 mM EGTA, and 5 mM glucose, pH 6.1). Fixed cells were permeabilized and blocked at RT for 40 min with cytoskeleton buffer supplemented to contain 0.25% saponin and 2% FBS. Cells were incubated at RT for 1 h with primary antibodies diluted 1:200 in cytoskeleton buffer supplemented to contain 0.25% saponin and 2% FBS. The following antibodies were used: mouse anti-non-muscle Myosin IIA monoclonal antibody [2B3] (Abcam; ab55456), rabbit anti-reovirus polyclonal antiserum specific for T3D, mouse 9GB5 anti-σ1 monoclonal antibody specific for T3D σ1, rabbit anti-LAMP-1 polyclonal antibody (Abcam; ab24170), rabbit VU219 anti-σ3 polyclonal antibody, mouse 8H1 anti-σ3 monoclonal antibody, and rabbit anti-reovirus polyclonal antiserum specific for T1L. Alexa Fluor–conjugated antibodies (Invitrogen) were used as secondary antibodies diluted 1:500 in cytoskeleton buffer supplemented to contain 0.25% saponin and 2% FBS. Cell nuclei were stained with 4′,6-diamidino-2-phenylindole (DAPI) (Sigma-Aldrich) diluted 1:200 in cytoskeleton buffer supplemented to contain 0.25% saponin and 2% FBS at RT for 20 min. Coverslips were mounted using Prolong-Gold (Life Technologies). Confocal microscopy images were acquired using a Leica TCS SP5 confocal multispectral microscope equipped with an HCX PL APO 63.0 X/1.4 NA oil objective and LAS X Life Science software (Leica Microsystems). Stimulated emission depletion (STED) analysis was conducted using a Leica TCS SP8 STED microscope equipped with an HC PL APO CS2 100 × /1.4 NA oil objective and LAS X software (Leica Microsystems).

For live-cell imaging microscopy, cells were cultivated on glass-bottom culture p35 plates (Ibidi). Total internal reflection fluorescence (TIRF) images were collected every 3 s for 10 min using a Leica DMi8 S widefield epifluorescence microscope equipped with a Hamamatsu Flash 4 sCMOS digital camera and an HC PL APO 100 × /1.47 oil objective. The TIRF module was equipped with a Hamamatsu W-View Gemini for simultaneous GFP/mCherry TIRF image acquisition. Images were processed using the LAS X software. HeLa cells cultivated on glass-bottom p35 culture plates (Ibidi) were transfected with the plasmids encoding T3D σ3-GFP [13] and pCMV-mCherry-MHC-IIA (gift from Venkaiah Betapudi [58], Addgene; #35687) using FuGene 6 (Promega, E2691) at a 3:1 FuGene 6: DNA ratio. At 24 h post-transfection, cell monolayers were uninfected or adsorbed with reovirus T1L M1-P208S at an MOI of 5 PFU/cell for 24 h. The videos were made collecting fluorescence images from 1 to 24 h post adsorption every 30 min using a Leica DMI6000B fluorescence microscope equipped with an HC PL FLUO 40 x/ 0.75 objective and LAS X software. Infected cells were identified by the presence of VFs, which appear by phase-contrast microscopy as dense and globular structures.

### Transmission electron microscopy

For conventional electron microscopy, cells were grown on sterile Thermanox Plastic Coverslips (Nunc) and fixed with a mixture of 4% paraformaldehyde and 1% glutaraldehyde in 0.4 M HEPES buffer, pH 7.4, at RT for 1 h. Cells were postfixed by incubation at 4°C for 1 h with a mixture of 1% osmium tetroxide and 0.8% potassium ferricyanide in water and dehydrated with increasing concentrations of acetone (50%, 70%, 90%, and twice in 100%) incubated at 4°C for 5 min each. Samples were processed for flat embedding in the epoxy resin EML-812 (TAAB Laboratories) by incubating at RT overnight with a 1:1 mixture of acetone and resin. Cells were infiltrated for 8 h in pure resin and polymerized at 60°C for 48 h. For embedding of purified lysosomes in epoxy resin, samples were fixed in 1% glutaraldehyde diluted in 0.4 M HEPES buffer, pH 7.4 and washed one time with HEPES. The lysosomes were collected by centrifugation at 21,000 x *g* at 4°C for 30 min. The supernatant was removed and the lysosomes resuspended in the smallest volume of HEPES buffer possible. An equal amount of 2% agar (Sigma-Aldrich; A5030) was added and mixed carefully and incubated on ice for 20 min to solidify. The solidified pellets were cut into small cubes and incubated with a mixture of 1% osmium tetroxide and 0.8% potassium ferricyanide in water at 4ºC for 1 h. The samples were washed three times with water and dehydrated with increasing concentrations of acetone (25%, 30%, 50%, 70%, 90%) at 4°C for 5 min and twice in 100% acetone at 4°C for 10 min. The lysosomes were incubated at RT overnight with a 1:1 mixture of acetone and resin in a rotating wheel. The samples were changed to pure resin, incubated at RT for 8 h in a rotating wheel, and polymerized in BEEM embedding capsules (TED PELLA, INC.; 130-B) at 60ºC for 48 h. Ultrathin (∼50–70 nm) oriented serial sections were obtained with a diamond knife using an UC6 ultramicrotome (Leica Microsystems), collected on uncoated 200-mesh copper grids (TAAB Laboratories), stained with saturated uranyl acetate and lead citrate, and imaged by transmission electron microscopy.

For negative staining of purified viral particles and lysosomes, 200-mesh copper grids with a film of 0.5% formvar in chloroform and coated with 3–4 nm of carbon were used. The formvar-carbon-coated grids were treated with a glow discharge of 25 mA for 15 s to render the surface of the grid hydrophilic, improving the sample attachment and homogenous distribution. The samples (6 μl) were incubated on the formvar-carbon side of the grid at RT for 2 min, blotted by removing the excess solution through touching the grid edge with a filter paper, and washed three times with deionized water. For purified viral particle samples, grids were incubated with 2% uranyl acetate for 40 s and blotted to dryness. Grids with the samples of purified lysosomes were fixed with 1% glutaraldehyde diluted in PBS at RT for 30 min, washed three times with PBS, incubated with 3% phosphotungstic acid (PTA) at RT for 40 s, and blotted to dryness.

Immunogold labeling of viral particles was performed using 0.5% formvar-carbon-coated 200-mesh copper grids treated with a glow discharge (25 mA, 15 s). The sample drops (6 μl) were incubated with the grids for 2 min, fixed with 3% paraformaldehyde at RT for 20 min and washed three times with PBS. The grids were incubated at RT with 50 mM NH_4_Cl in PBS for 5 min to quench free aldehyde groups, blocked with 1% BSA for 5 min, and incubated with the primary antibody diluted in 1% BSA at RT for 15–30 min. The following antibodies diluted 1:40 were used: mouse anti-non-muscle Myosin IIA monoclonal antibody [2B3] (Abcam; ab55456), mouse anti-β-actin monoclonal antibody (Sigma-Aldrich; A5441), rabbit anti-reovirus polyclonal antiserum specific for T1L, mouse 5C6 anti-σ1 monoclonal antibody specific for T1L σ1, and mouse J2 anti-dsRNA IgG2a monoclonal antibody (English and Scientific consulting; 10010500). The grids were washed three times with 1% BSA and incubated at RT for 30 min with a secondary antibody conjugated with 10-nm colloidal gold particles (BB International) diluted 1:100 in 1% BSA. The samples were washed three times with PBS and five times with deionized water and allowed to dry for 30 min. To negative stain the samples, the grids were incubated with 2% uranyl acetate for 40 s and allowed to dry before imaging in the electron microscope.

For immunolabeling thawed cryosections, HBMECs were fixed at 24 h post-adsorption with 4% paraformaldehyde in PHEM buffer, pH 7.2 (60 mM Pipes, 25 mM HEPES, 10 mM EGTA, and 2 mM MgCl_2_) at RT for 2 h. Fixed monolayers were incubated with 50 mM NH_4_Cl to quench free aldehyde groups. Cells were removed from the plastic plate using a rubber policeman and pelleted in a 1.5-ml Eppendorf tube. Pellets were embedded in 12% gelatin (TAAB Laboratories) in PBS and solidified on ice for 15 min. Cell pellets were sectioned into cubes of 1 mm^3^ and infiltrated at 4°C overnight with 2.3 M sucrose in PBS. Blocks were mounted on metal pins and frozen in liquid nitrogen. Ultrathin cryosections (50 – 100 nm) were obtained at -120°C with a diamond knife using an FC6 ultramicrotome (Leica Microsystems). Sections were collected into a mixture of 2% methylcellulose in water and 2.3 M sucrose in PBS (1:1) and transferred after thawing to 200-mesh grids with a carbon-coated formvar film. Before labeling with antibodies, grids were incubated in PBS in a humid chamber at 37°C for 25 min. Free aldehydes were quenched with 50 mM NH_4_Cl (five times for 2 min each) before incubation with 1% BSA at RT for 5 min for blocking. Cryosections were labeled with the mouse anti-non-muscle Myosin IIA monoclonal antibody [2B3] (Abcam; ab55456) diluted 1:40 in 1% BSA at RT for 1 h. After washes with 0.1% BSA (five times for 2 min) and 1% BSA (once for 5 min), grids were incubated at RT for 30 min with a secondary antibody conjugated with 10-nm colloidal gold particles (BB International) and diluted 1:100 in 1% BSA. Cryosections were washed with 0.1% BSA (two times for 2 min) and PBS (three times for 2 min) before post-fixation with 1% glutaraldehyde in PBS at RT for 5 min. After washing with water (nine times for 2 min), grids were incubated with a mixture of uranyl acetate and methylcellulose (9:1) on ice for 5 min. Grids were removed from the methyl cellulose–uranyl acetate solution using a 3.5-mm-diameter wire loop. The excess liquid from the loop was removed and grids were allowed to dry. The grids were studied using a JEOL JEM 1400 Flash electron microscope operating at 100 kV equipped with a Gatan OneView digital and direct detection camera.

### TEM and STEM tomography

Infected HBMECs were fixed with 1% glutaraldehyde in 0.4 M HEPES buffer, pH 7.4 before high-pressure freezing and freeze-substitution. Cell pellets were washed with PBS to remove fixative and frozen with fetal calf serum as cryo protectant in 3mm sample holder type A in the 200-micron cavity with a Leica EM ICE (Leica microsystems). Freeze substitution was conducted in an AFS2 (Leica microsystems) with 1% osmium tetroxide and with 0.1% uranyl acetate in acetone containing 5% water and 2% methanol. Following completion of the substitution protocol, samples were gradually infiltrated with epon (TAAB laboratories) and polymerized by incubating at 60°C for 48 h.

Embedded samples were cut with a nominal thickness of 150 or 200 nm using an UC6 microtome (Leica microsystems) and collected on Formvar-carbon-coated 100 mesh copper grids (Gilder). BSA-gold (fiducials) of 10 nm (EMS) was applied on both sides of the sections, and poststaining was performed with 4% aqueous uranyl acetate followed by Reynold´s lead citrate. For transmission electron tomography, dual-axis electron tomograms were collected on a Talos F200C transmission electron microscope (Thermo Fisher Scientific) operated at 200 kV and equipped with a Ceta camera. Tilt series were collected at a magnification of 36,000x with a pixel size of 0.41 nm over a range from −60° to +60° with an angular increment of 1°. For scanning transmission electron tomography, single-axis tomograms were collected on a Talos F200C microscope operated at 200 kV in nano-probe mode with a bright-field detector (Thermo Fisher Scientific) at a magnification of 87,000x and an image size of 2,048 by 2,048 pixels. Tilt series were collected from −60° to +60° with an angular increment of 1°. In the present work, we improved our 3D tomography study to obtain higher resolution and more details of the structures because: 1) sections were thinner (150-200 nm vs 300 nm); 2) we applied a lower increment for the tilt series (1 degree vs 1.5 degree); 3) sections were stained with both uranyl acetate and lead citrate, not with uranyl acetate only and 4) fiducials were applied on both sides of the grids, allowing for better alignment of the tilt series. Points 1 – 3 contribute to a better S/N ratio. Points 1 and 2 contribute to better resolution. Point 4 allows a better alignment of the tilt series after acquisition. Additionally, images were acquired at higher magnification (11,500x *vs* 36,000x) with a Ceta camera (CMOS) instead of an Eagle camera (CCD).

Projection alignment and image processing of the tomograms was conducted using the IMOD program [60]. Visualization and final segmentation were conducted using Amira software. Before segmentation, a subset of tomograms was subjected to “denoising” by a Gaussian filter application [61–64]. A total of forty tomograms showing different reovirus egress compartments were recorded.

### Quantification and statistical analysis

Experiments were conducted with a minimum of three independent replicates. Data presented in the text are expressed as the mean ± SEM or the relative mean ± SD. Statistical significance was determined using two-sample unequal variance t test with one- or two-tailed distribution (α = 0.05). Graphs were prepared and statistical analyses were conducted using Microsoft Excel and GraphPad Prism9 software.

## Supporting information

S1 Fig Expression of myosin-9 and actin in mock- and reovirus-infected cells. HBMECs were either mock infected or infected with reovirus T3D at an MOI of 1 PFU/cell and incubated for 24 h. Myosin-9, actin, and GAPDH levels were determined by immunoblotting (representative of three independent experiments). Signal intensity of bands corresponding to myosin-9, actin, and GAPDH were quantified. The *p*-values (one- and two-tailed, respectively) were calculated using student’s *t*-test.
(TIF)

S2 Fig Myosin-9 interaction with reovirus proteins. HBMECs were infected with reovirus T3D at an MOI of 500 PFU/cell and incubated for 24 h. Cells were processed for co-immunoprecipitation. Myosin-9 interactions with reovirus proteins were assessed using SDS-PAGE and immunoblotting with antibody specific for σ3. Immunoblot is representative of two independent experiments.
(TIF)

S3 Fig Proteomic analysis of intracellular reovirus purified from HBMECs and L929 cells reveals similar cellular factors. Cells were infected with reovirus T1LM1-P208S at an MOI of 5 PFU/cell and incubated for 72 h. Extracellular virions were isolated and prepared for LC-MS. Proteins were identified using the MASCOT database. Potential candidates with a Q-value < 0.01 and at least two peptides identified in all three replicates were selected for analysis. (A and B) The top 10 candidates from the proteomic analysis of reovirus virions purified from HBMECs and L929 cells were ranked by their Sum PEP Scores. The graphs show the biological processes using Gene Ontology (GO). (C) Functional protein-protein interaction networks of the protein candidates identified in the STRING analyses.
(TIF)

S4 Fig Electron microscopy of reovirus purified from L929 cells. Cells were infected with reovirus T1LM1-P208S at an MOI of 5 PFU/cell and incubated for 72 h. Intracellular virions were isolated and processed for negative staining and electron microscopy. (A) Low-magnification view of purified virions. Scale bar, 200 nm. (B) Higher-magnification view of purified virions. No filaments are observed. Scale bar, 100 nm.
(TIF)

S5 Fig Immunogold labeling and transmission electron microscopy detecting reovirus proteins on the virion surface and dsRNA associated with virions. HBMECs and L929 cells were infected with reovirus T1LM1-P208S at an MOI of 5 PFU/cell and incubated for 72 h. Intracellular virions were isolated and labeled for (A) reovirus (rabbit anti-reovirus polyclonal antiserum specific for T1L), (B) σ1 (mouse 5C6 anti-σ1 monoclonal antibody specific for T1L σ1) and (C) dsRNA (mouse J2 anti-dsRNA IgG2a monoclonal antibody) using secondary antibodies conjugated with 10-nm gold particles, and imaged using a transmission electron microscope. (A) Reovirus proteins and (B) reovirus protein σ1 are detected on the surface of the virions (arrows). (C) Purified virions were disrupted by freezing and thawing before immunogold labeling (arrows). EM images show open particles with dsRNA-labeled structures. Scale bars, 100 nm.
(TIF)

S6 Fig Confocal microscopy, STED super-resolution microscopy, and TIRF super-resolution live-cell microscopy of myosin-9 redistribution in reovirus-infected cells. (A-E) HBMECs were either uninfected or infected with reovirus T1LM1-P208S at an MOI of 1 PFU/cell, incubated for 18 h, and processed for immunofluorescence and confocal microscopy. Cells were stained with antibodies specific for myosin-9 (red) and σ3 (green). (A) Myosin-9 in mock-infected cells has a cytoplasmic distribution. (B, D, and E) low- and high-magnification views of reovirus-infected cells showing myosin-9 in fibers (arrows) near VFs (asterisks). Scale bars, 10 μm. (C) Super-resolution STED microscopy showing association of myosin-9 (red, arrowheads) and reovirus (green) at the cell periphery. Scale bar, 3 μm. (F-H) TIRF super-resolution live-cell microscopy of myosin-9 in reovirus-infected cells. HeLa cells were transfected with plasmids encoding σ3-GFP and myosin-9-mCherry for 24 h and infected with reovirus T1L M1-P208S at an MOI of 5 PFU/cell. Cells were incubated for 24 h and imaged using TIRF microscopy to visualize basal reovirus egress zones. Myosin-9 and σ3 were visualized with the mCherry and GFP fusion proteins (red and green, respectively). Myosin-9 and σ3 (arrowheads) colocalize at the plasma membrane. Scale bars, 10 μm.
(TIF)

S7 Fig Immunogold labeling and transmission electron microscopy of myosin-9 in reovirus-infected cells. HBMECs were either uninfected or infected with reovirus T3D at an MOI of 1 PFU/cell and cryo-sectioned using the Tokuyasu method at 24 h post-infection. Cryosections were immunogold labeled for myosin-9 and a secondary antibody conjugated with 10-nm gold particles. (A) Myosin-9 signal (arrowheads) is located in the cytoplasm of uninfected cells. N, nucleus; pm, plasma membrane. Scale bars, 200 nm. (B) Myosin-9 signal (black arrowheads) is located in VFs of reovirus-infected cells and inside membranous compartments (asterisks) compatible with lysosomes adjacent to VFs. Myosin-9 signal (black arrowheads) is detected in the same membranous compartments as viral particles (white arrowheads) compatible with SOs or MCs. mi, mitochondrion. Scale bars, 200 nm.
(TIF)

S8 Fig Efficiency of myosin-9 siRNA silencing in mock- and reovirus-infected cells. HBMECs were non-transfected or transfected with scramble or myosin-9 siRNAs and either uninfected or infected with reovirus T3D at an MOI of 5 PFU/cell at 24 h post-transfection. Cells were incubated for 24 h. Myosin-9 and GAPDH levels were determined by immunoblotting (representative of three independent experiments). Myosin-9 silencing in uninfected and reovirus-infected cells was quantified.
(TIF)

S9 Fig Confocal microscopy analysis of myosin-9 and reovirus signal distributions in infected cells. HBMECs were non-transfected or transfected with scramble or myosin-9 siRNAs and infected with reovirus T3D at an MOI of 5 PFU/cell at 24 h post-transfection. Cells were incubated for 24 h and imaged using confocal microscopy. (A and B) Immunofluorescence images of the cytoplasmic distribution of myosin-9 and reovirus in infected cells stained with antibodies specific for myosin-9 (red) and reovirus (green). Nuclei were labeled with DAPI (blue). In cells transfected with myosin-9 siRNA, the remaining myosin-9 redistributes and forms filaments in some reovirus-infected cells. The fluorescent reovirus signal concentrates primarily inside factories. Higher magnification views of selected areas are shown in B. Scale bars, 25 µm.
(TIF)

S10 Fig Effects of myosin-9 silencing on reovirus protein expression. HBMECs were non-transfected or transfected with scramble or myosin-9 siRNAs and either uninfected or infected with reovirus T3D at an MOI of 5 PFU/cell at 24 h post-transfection. Cells were incubated for 24 h. (A) Reovirus structural proteins µ1, µ1C, and σ3 were detected by immunoblotting using T3D-specific antiserum. Tubulin was detected using tubulin-specific antibody. (B) Reovirus nonstructural proteins µNS and σNS were detected using chicken polyclonal and rabbit polyclonal antibodies, respectively. GAPDH was used as loading control. Immunoblots are representative of three independent experiments. Signal intensity of reovirus proteins µ1, µ1C, σ3, µNS, and σNS were quantified ± standard deviation (SD) for all the experiments. The *p*-values (one- and two-tailed, respectively) were calculated using student’s *t*-test.
(TIF)

S11 Fig Myosin-9 is not required for reovirus disassembly in the endocytic pathway. HBMECs were non-transfected or transfected with scramble or myosin-9 siRNAs and infected with reovirus T1LM1-P208S virions or infectious subvirion particles (ISVPs) at an MOI of 5 PFU/cell at 24 h post-transfection. Cells were incubated for 18 h and processed for immunofluorescence. (A) Schematic of cell entry by receptor-mediated endocytosis of virions or ISVPs. Prepared using BioRender. Tenorio, R. (2025) https://BioRender.com/oeo07s5. (B) Representative confocal micrographs of cells immunostained using a rabbit polyclonal antiserum specific for reovirus (green). Nuclei were labeled with DAPI (blue). Scale bars, 50 µm. (C) The percentage of infected cells was determined by enumerating reovirus-infected cells in immunofluorescence images and presented ± SEM of three independent experiments. Significance was determined by unpaired two-tailed student’s *t*-test; ** *p* < 0.01 and **** *p* < 0.0001.
(TIF)

S12 Fig High magnification views of the effect of myosin-9 silencing on the distribution of lysosomes. HBMECs were non-transfected or transfected with scramble or myosin-9 siRNAs and either uninfected or infected with reovirus T3D at an MOI of 5 PFU/cell at 24 h post-transfection. Cells were incubated for 24 h and imaged using confocal microscopy. Immunofluorescence images show cells stained for reovirus capsid protein σ1 (green), LAMP1 (red), and DAPI to label nuclei (blue). (A) In non-transfected or scrambled siRNA-transfected cells, LAMP1 staining (red) was distributed diffusely in the cytoplasm. In myosin-9 siRNA-transfected cells, LAMP1 staining concentrated in the perinuclear region (arrows). (B and C) In reovirus-infected cells, reovirus σ1 staining distributed in the cytoplasm in non-transfected or scrambled siRNA-transfected cells and inside reovirus factories (asterisks) in cells in which myosin-9 expression was silenced (arrowheads). Aggregated lysosomes located near the VFs are indicated with arrows. Scale bars, 10 µm.
(TIF)

S13 Fig Transmission electron microscopy of the effect of myosin-9 silencing on the distribution of lysosomes in mock-infected cells. HBMECs were non-transfected or transfected with scramble or myosin-9 siRNAs. At 24 h post-transfection, cells were processed for electron microscopy. (A, D and G) Ultrathin sections of low-magnification views of cells non-transfected or transfected with scramble and myosin-9 siRNAs are shown. Lysosomes (dashed box) accumulate in a discrete area of the cytoplasm in a cell in which myosin-9 has been silenced (g). N, nucleus. Scale bars, 1 µm. (B, E and H) Higher-magnification views of the cytoplasm with lysosomes indicated (asterisks). Scale bars, 500 nm. (C, F and I) Lysosome morphology and structure. Scale bars, 200 nm.
(TIF)

S14 Fig Transmission electron microscopy of lysosome recruitment to VFs in infected cells. HBMECs were non-transfected or transfected with scramble siRNAs and infected with reovirus T3D at an MOI of 5 PFU/cell at 24 h post-transfection. Cells were incubated for 24 h and processed for electron microscopy. (A and C) Low-magnification views of cells non-transfected or transfected with scramble siRNA and infected with reovirus. Lysosomes (asterisks) are adjacent to VFs in the cytoplasm. Scale bars, 1 µm. (B and D) VFs and lysosomes at higher magnification. Viral particles (arrowheads) are seen inside a lysosome in a scramble siRNA-transfected cell. N, nucleus. Scale bars, 200 nm.
(TIF)

S15 Fig Confocal and electron microscopy of the effects of myosin-9 inhibition with blebbistatin on the recruitment of mature virions to the egress machinery. HBMECs were either uninfected or infected with reovirus T1LM1-P208S at an MOI of 1 PFU/cell. At 17 h post-infection, cells were incubated with 50 μM blebbistatin or DMSO control for 1 h and processed for confocal and electron microscopy. (A) Cells were immunostained with antibodies specific for reovirus σ3 protein (green) and LAMP1 (red). Nuclei were labeled with DAPI (blue). Images show reovirus σ3 protein distribution in reovirus-infected cells in the absence and presence of blebbistatin. In untreated infected cells, σ3 is distributed in the cytoplasm and inside VFs (asterisks). Lysosomes (white arrows) marked by LAMP1 are mostly adjacent to factories in the perinuclear region. In blebbistatin-treated cells, cytoplasmic σ3 disappears, and the protein concentrates in VFs (asterisks). The LAMP1 signal is weak in cells treated with blebbistatin. Scale bars, 10 μm. (B) EM images of infected cells in the absence (left) or presence of (right) blebbistatin treatment. In untreated cells, the SO is filled with mature virions near a VF. In blebbistatin-treated cells, the SO contains mostly immature and empty viral particles near a VF. (C) Representative examples of membranous carriers (MCs) close to the plasma membrane (pm) are shown. In untreated infected cells (left), MCs contain mature virions. In cells treated with blebbistatin (right), MCs contain mostly empty viral particles. (D) Higher-magnification images of MCs presented in C. Mature virions (arrows) in untreated cells (left) are electron dense compared with the empty capsids (black arrowheads) and immature virions (white arrowheads) observed in the cell treated with blebbistatin (right). Scale bars, 200 nm.
(TIF)

S1 Table List of proteins identified in Figs 3 and 4, including their full names and UniProt accession numbers. (DOCX)

S1 Video Live-cell microscopy of myosin-9-mCherry expression in uninfected control cell. HeLa cells were transfected with plasmids encoding σ3-GFP and myosin-9-mCherry for 24 h. Fluorescence images were collected every 30 minutes using a 40 x objective. No σ3-GFP signal was observed in uninfected cells, whereas myosin-9-mCherry (red) showed a diffuse cytoplasmic signal.
(MP4)

S2 Video Live-cell microscopy of myosin-9-mCherry expression in reovirus-infected cells. HeLa cells were transfected with plasmids encoding σ3-GFP and myosin-9-mCherry. At 24 h post-transfection, cells were infected with T1LM1-P208S at an MOI of 5 PFU/cell. Fluorescence images were collected every 30 minutes using a 40 x objective. No σ3-GFP signal was observed. In reovirus-infected cells, the myosin-9-mCherry signal was concentrated in discrete areas.
(MP4)

S3 Striking image. (DOCX)
