# Supplementary figures and images for: Myosin-9 is required for lysosome-mediated nonlytic reovirus egress
Source: PLoS Pathog. 2025 Oct 14;21(10):e1013597. doi: 10.1371/journal.ppat.1013597 (PMC12543285; doi:10.1371/journal.ppat.1013597)

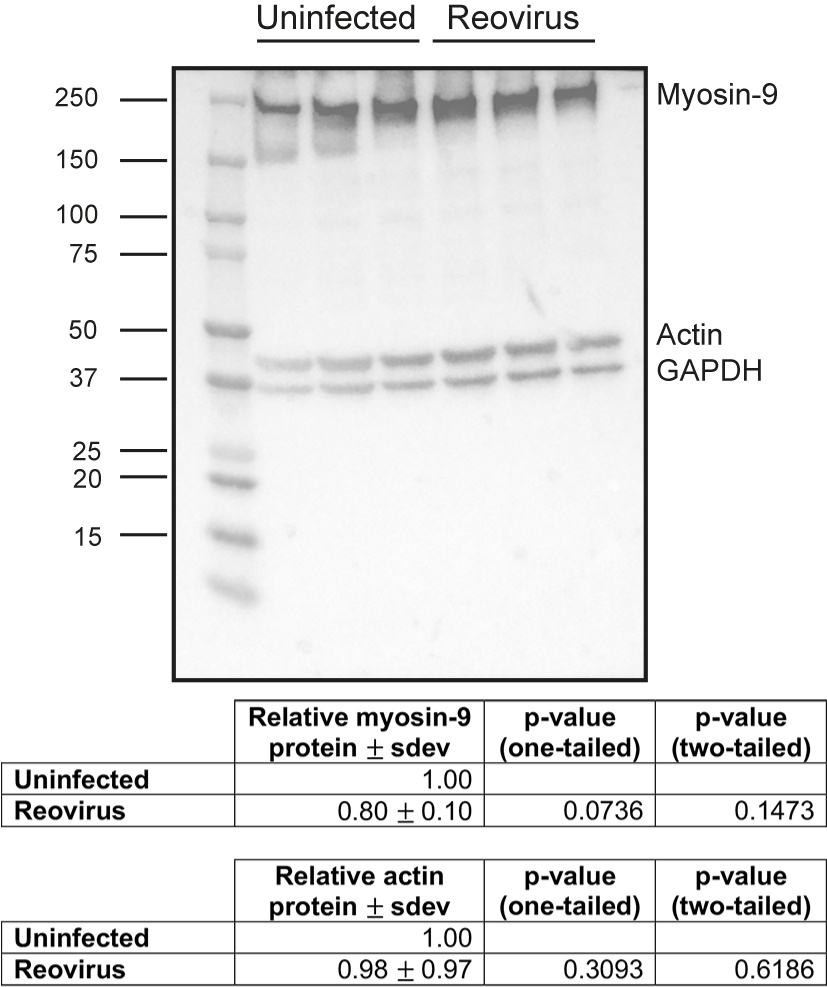

Supplement: S1 Fig — HBMECs were either mock infected or infected with reovirus T3D at an MOI of 1 PFU/cell and incubated for 24 h. Myosin-9, actin, and GAPDH levels were determined by immunoblotting (representative of three independent experiments). Signal intensity of bands corresponding to myosin-9, actin, and GAPDH were quantified. The p-values (one- and two-tailed, respectively) were calculated using student’s t-test. (TIF) [file ppat.1013597.s001.tif]

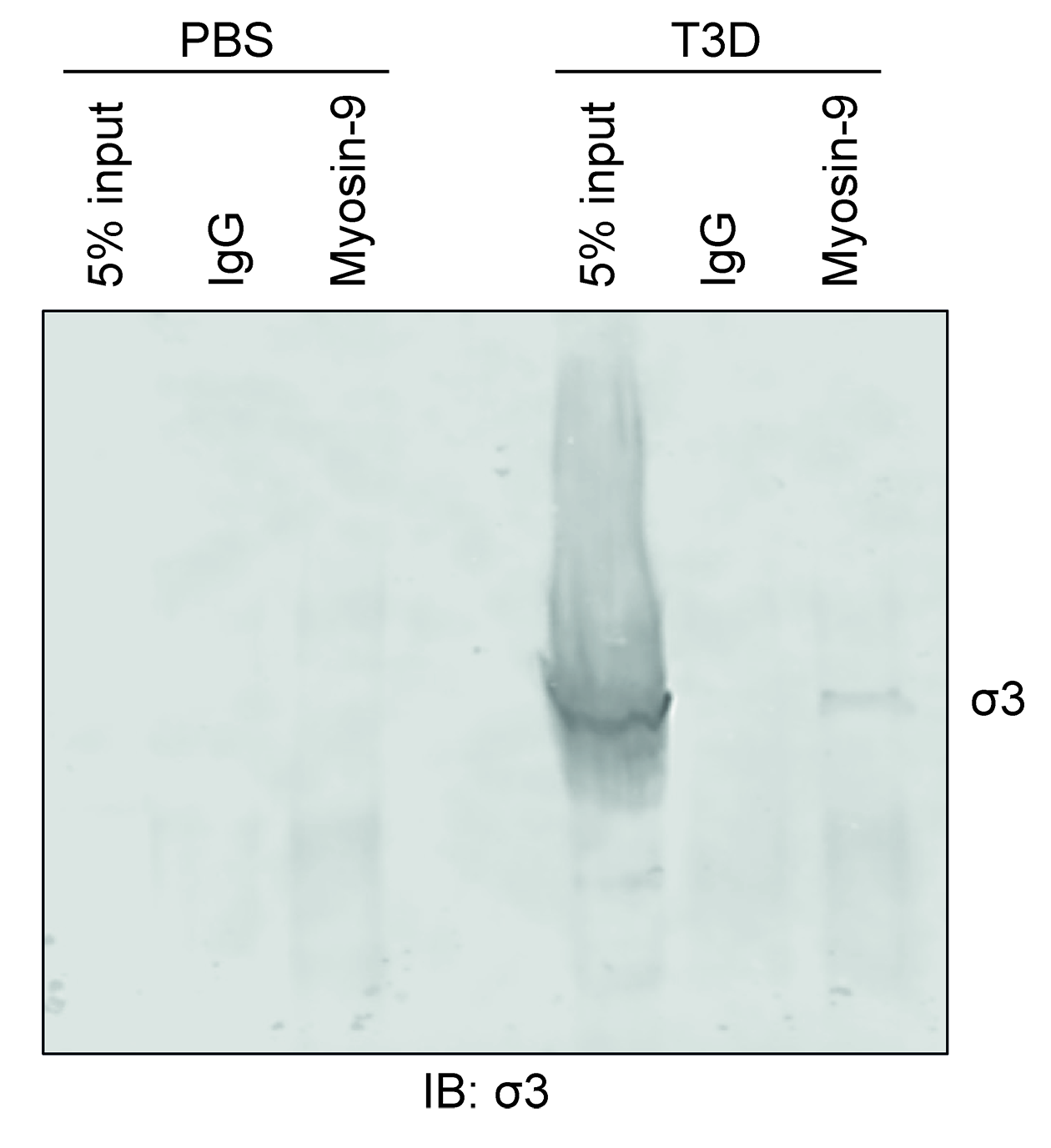

Supplement: S2 Fig — HBMECs were infected with reovirus T3D at an MOI of 500 PFU/cell and incubated for 24 h. Cells were processed for co-immunoprecipitation. Myosin-9 interactions with reovirus proteins were assessed using SDS-PAGE and immunoblotting with antibody specific for σ3. Immunoblot is representative of two independent experiments. (TIF) [file ppat.1013597.s002.tif]

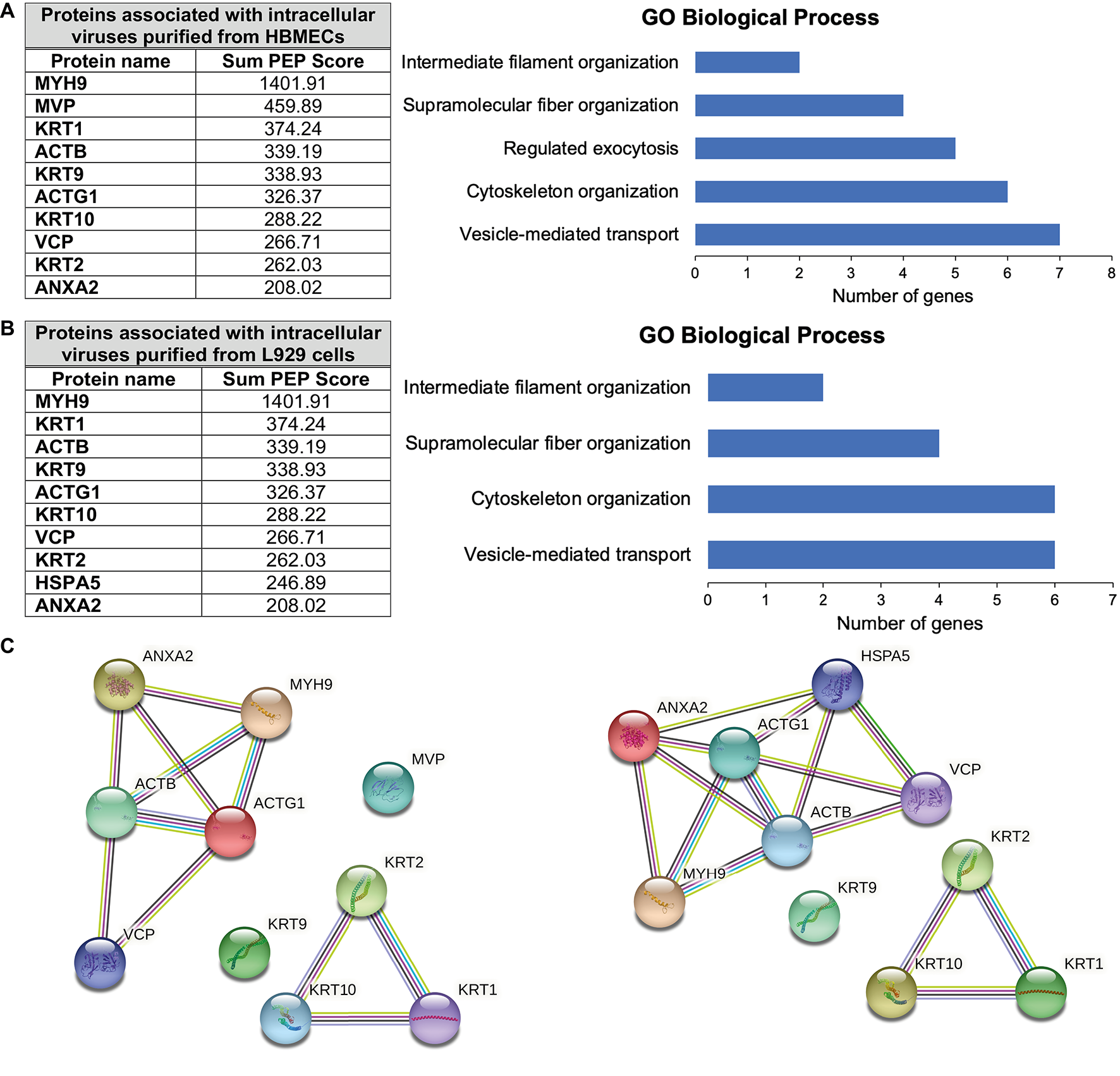

Supplement: S3 Fig — Cells were infected with reovirus T1LM1-P208S at an MOI of 5 PFU/cell and incubated for 72 h. Extracellular virions were isolated and prepared for LC-MS. Proteins were identified using the MASCOT database. Potential candidates with a Q-value < 0.01 and at least two peptides identified in all three replicates were selected for analysis. (A and B) The top 10 candidates from the proteomic analysis of reovirus virions purified from HBMECs and L929 cells were ranked by their Sum PEP Scores. The graphs show the biological processes using Gene Ontology (GO). (C) Functional protein-protein interaction networks of the protein candidates identified in the STRING analyses. (TIF) [file ppat.1013597.s003.tif]

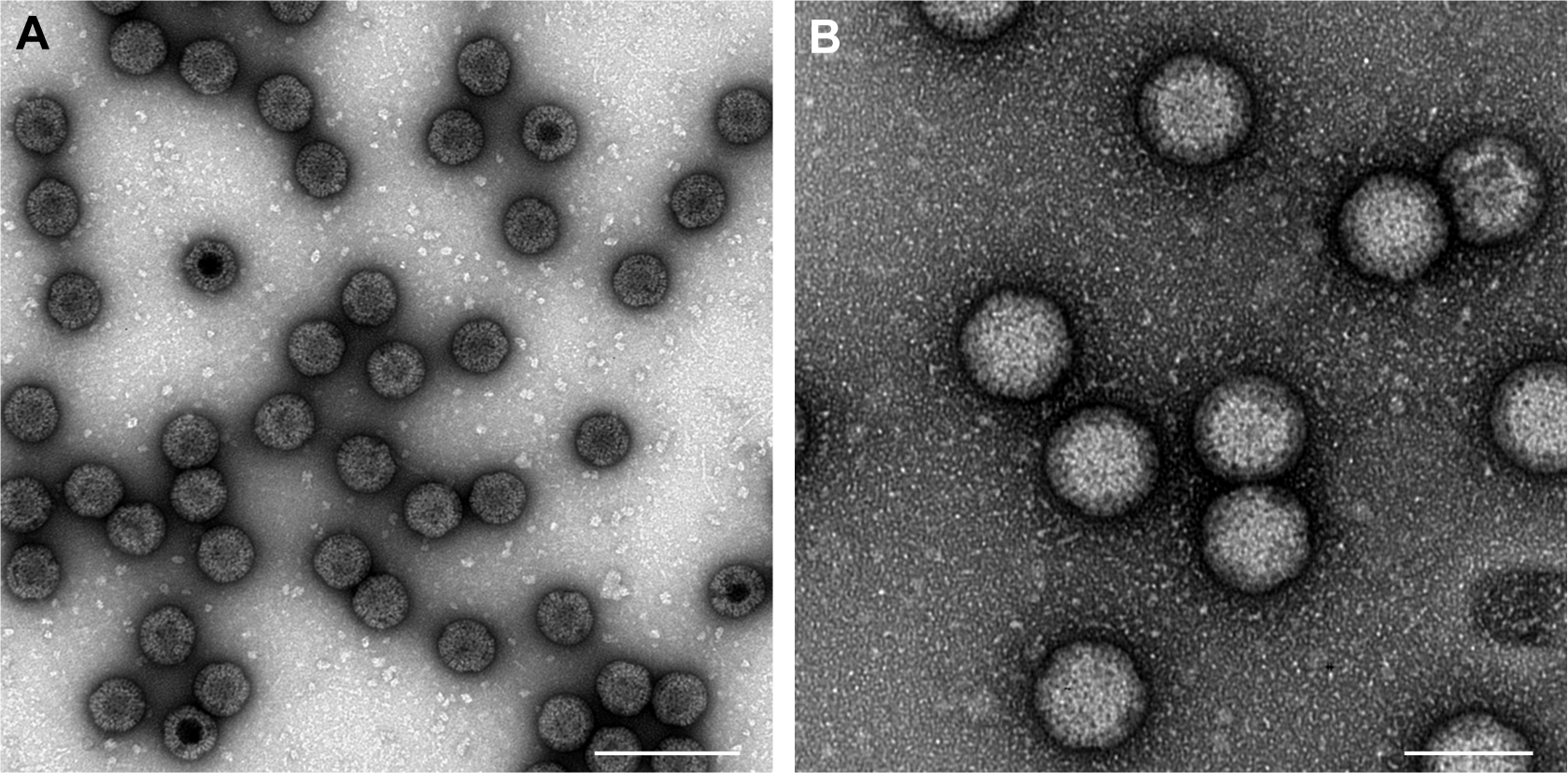

Supplement: S4 Fig — Cells were infected with reovirus T1LM1-P208S at an MOI of 5 PFU/cell and incubated for 72 h. Intracellular virions were isolated and processed for negative staining and electron microscopy. (A) Low-magnification view of purified virions. Scale bar, 200 nm. (B) Higher-magnification view of purified virions. No filaments are observed. Scale bar, 100 nm. (TIF) [file ppat.1013597.s004.tif]

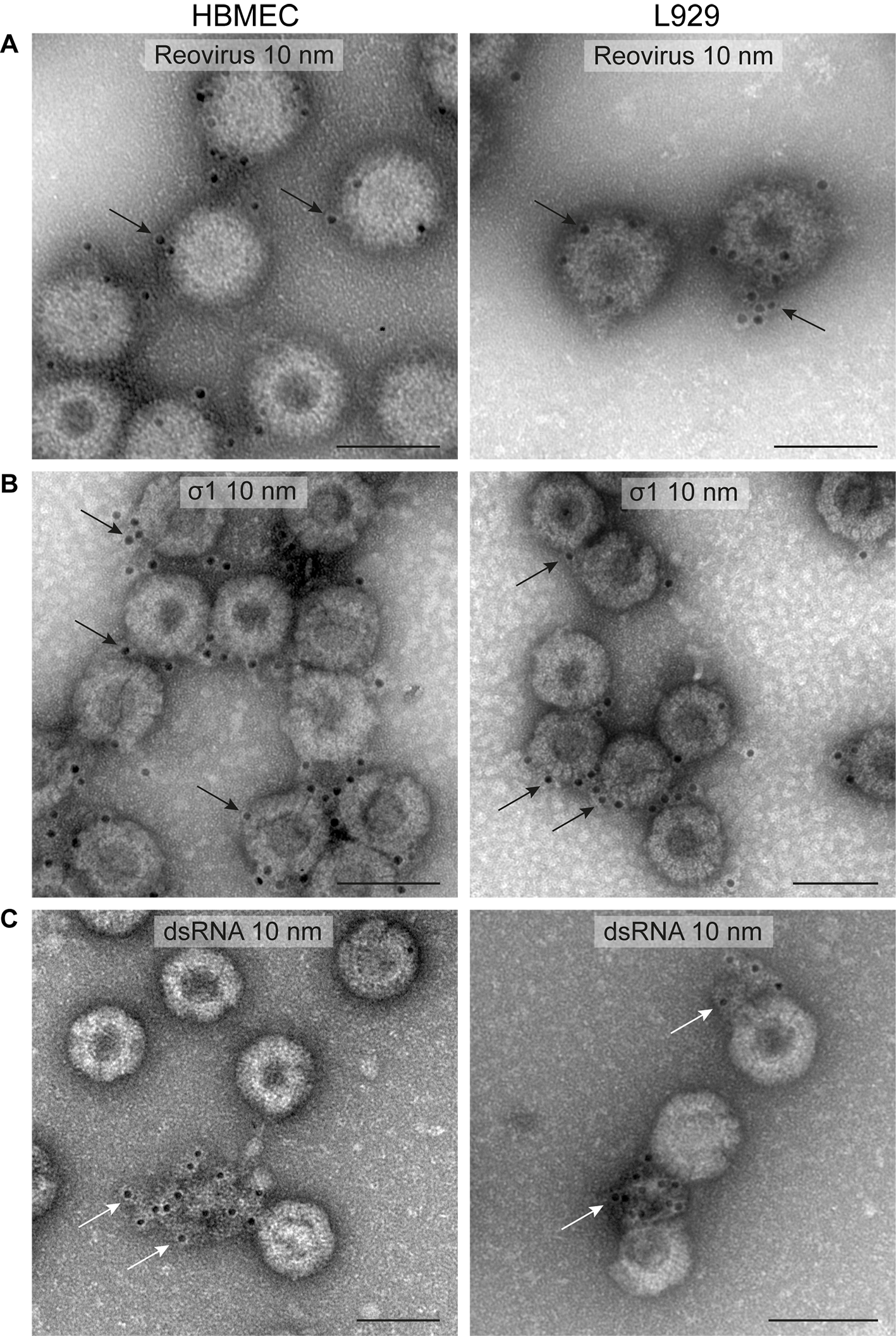

Supplement: S5 Fig — HBMECs and L929 cells were infected with reovirus T1LM1-P208S at an MOI of 5 PFU/cell and incubated for 72 h. Intracellular virions were isolated and labeled for (A) reovirus (rabbit anti-reovirus polyclonal antiserum specific for T1L), (B) σ1 (mouse 5C6 anti-σ1 monoclonal antibody specific for T1L σ1) and (C) dsRNA (mouse J2 anti-dsRNA IgG2a monoclonal antibody) using secondary antibodies conjugated with 10-nm gold particles, and imaged using a transmission electron microscope. (A) Reovirus proteins and (B) reovirus protein σ1 are detected on the surface of the virions (arrows). (C) Purified virions were disrupted by freezing and thawing before immunogold labeling (arrows). EM images show open particles with dsRNA-labeled structures. Scale bars, 100 nm. (TIF) [file ppat.1013597.s005.tif]

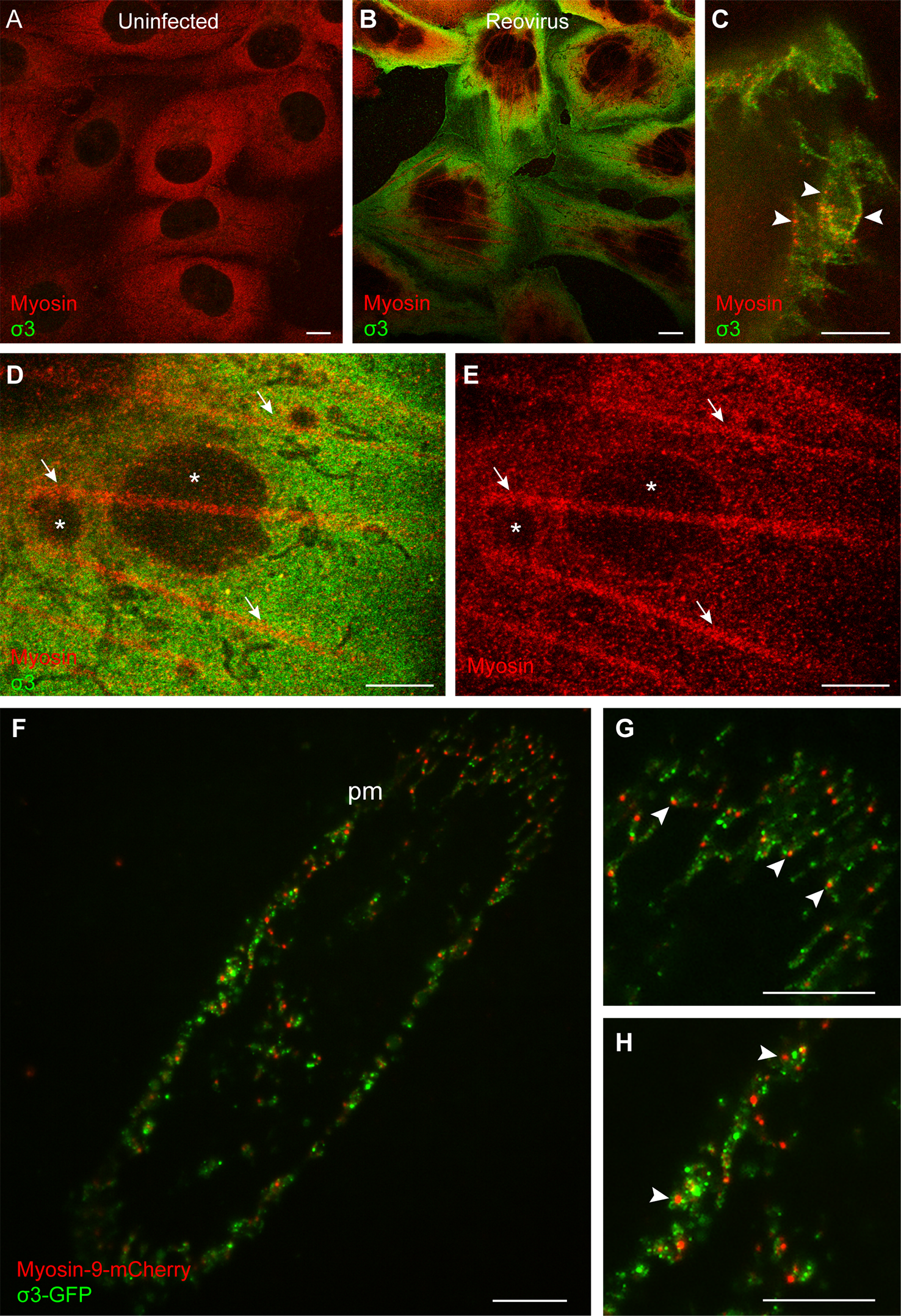

Supplement: S6 Fig — (A-E) HBMECs were either uninfected or infected with reovirus T1LM1-P208S at an MOI of 1 PFU/cell, incubated for 18 h, and processed for immunofluorescence and confocal microscopy. Cells were stained with antibodies specific for myosin-9 (red) and σ3 (green). (A) Myosin-9 in mock-infected cells has a cytoplasmic distribution. (B, D, and E) low- and high-magnification views of reovirus-infected cells showing myosin-9 in fibers (arrows) near VFs (asterisks). Scale bars, 10 μm. (C) Super-resolution STED microscopy showing association of myosin-9 (red, arrowheads) and reovirus (green) at the cell periphery. Scale bar, 3 μm. (F-H) TIRF super-resolution live-cell microscopy of myosin-9 in reovirus-infected cells. HeLa cells were transfected with plasmids encoding σ3-GFP and myosin-9-mCherry for 24 h and infected with reovirus T1L M1-P208S at an MOI of 5 PFU/cell. Cells were incubated for 24 h and imaged using TIRF microscopy to visualize basal reovirus egress zones. Myosin-9 and σ3 were visualized with the mCherry and GFP fusion proteins (red and green, respectively). Myosin-9 and σ3 (arrowheads) colocalize at the plasma membrane. Scale bars, 10 μm. (TIF) [file ppat.1013597.s006.tif]

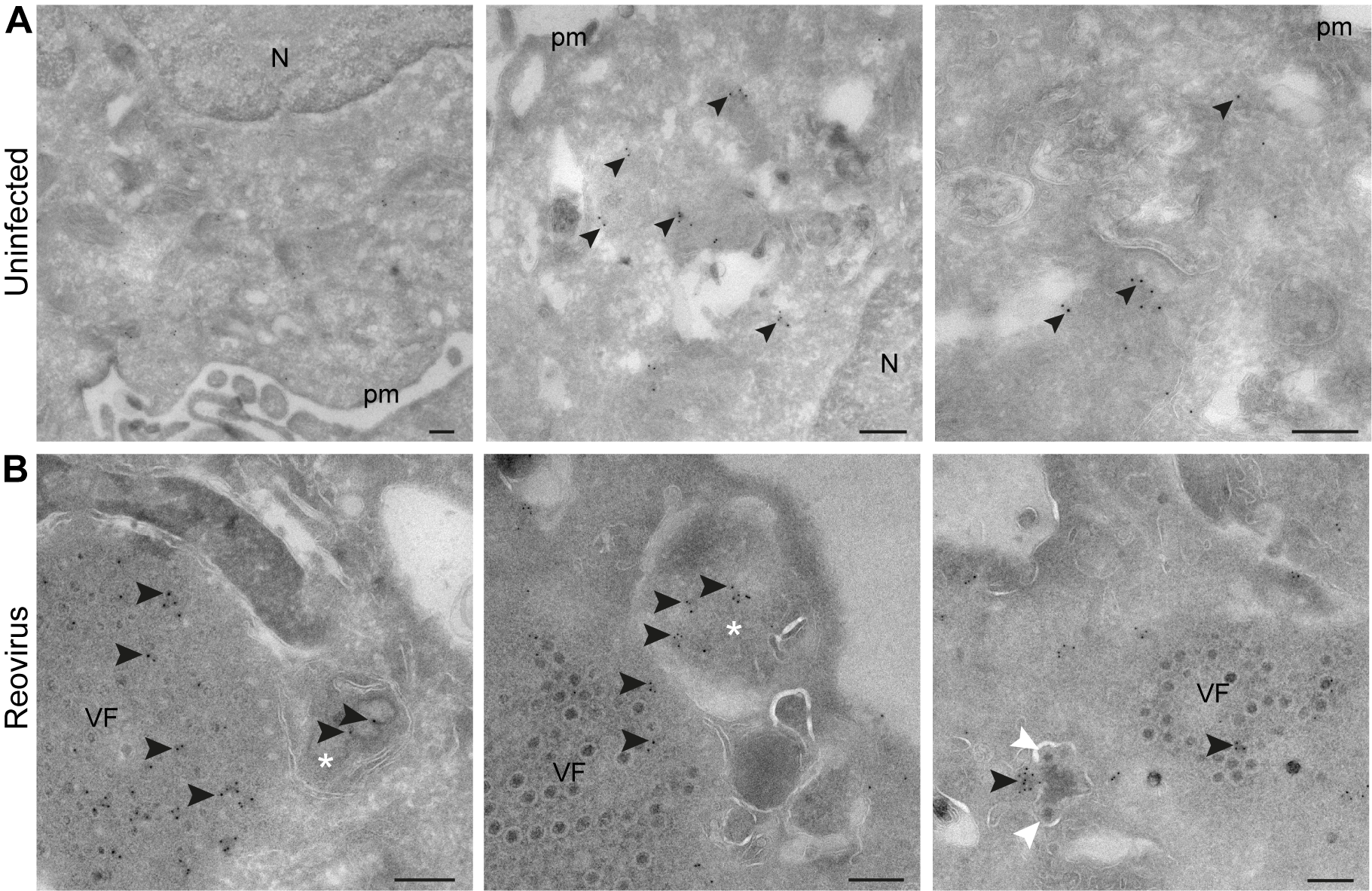

Supplement: S7 Fig — HBMECs were either uninfected or infected with reovirus T3D at an MOI of 1 PFU/cell and cryo-sectioned using the Tokuyasu method at 24 h post-infection. Cryosections were immunogold labeled for myosin-9 and a secondary antibody conjugated with 10-nm gold particles. (A) Myosin-9 signal (arrowheads) is located in the cytoplasm of uninfected cells. N, nucleus; pm, plasma membrane. Scale bars, 200 nm. (B) Myosin-9 signal (black arrowheads) is located in VFs of reovirus-infected cells and inside membranous compartments (asterisks) compatible with lysosomes adjacent to VFs. Myosin-9 signal (black arrowheads) is detected in the same membranous compartments as viral particles (white arrowheads) compatible with SOs or MCs. mi, mitochondrion. Scale bars, 200 nm. (TIF) [file ppat.1013597.s007.tif]

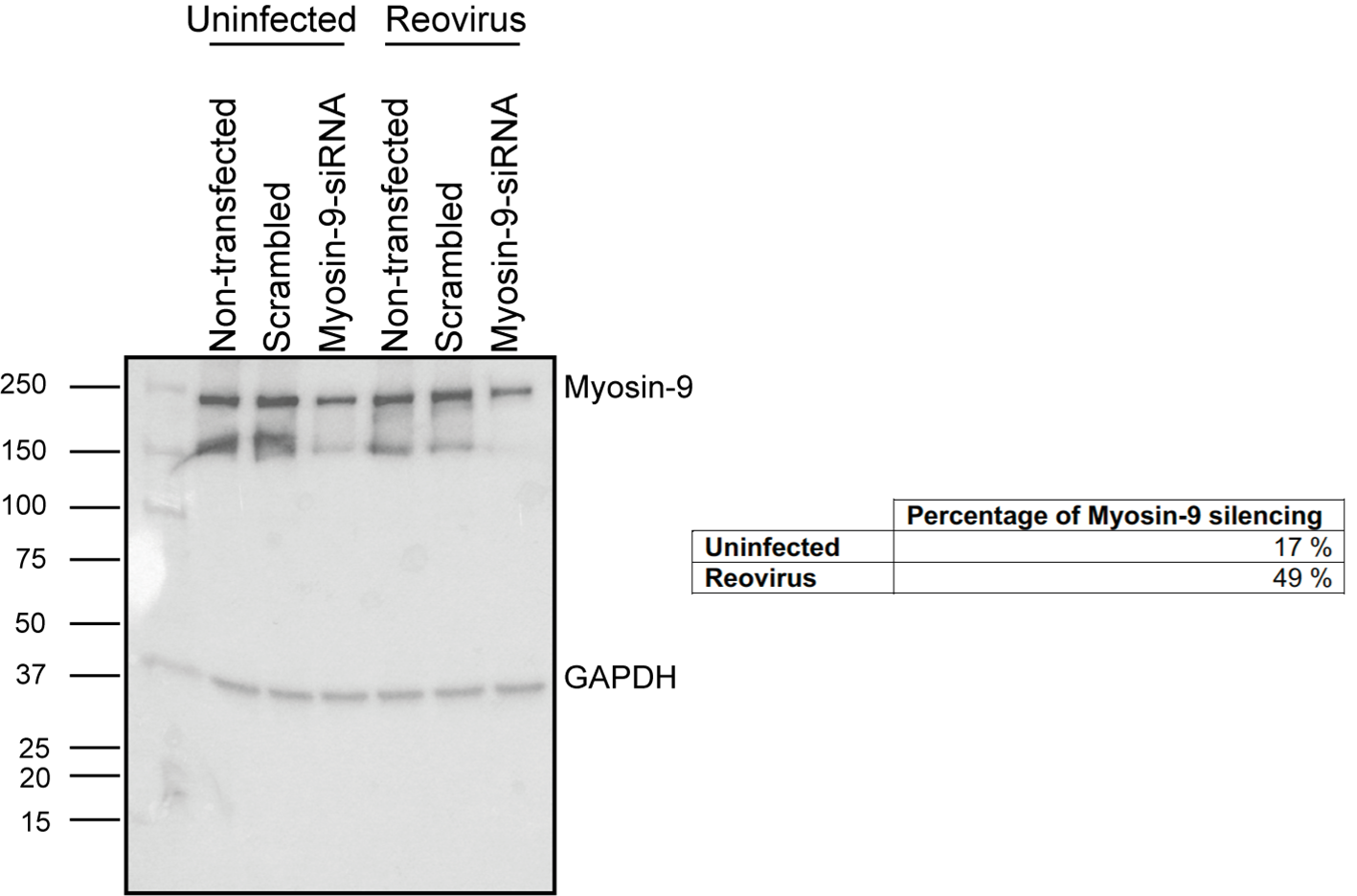

Supplement: S8 Fig — HBMECs were non-transfected or transfected with scramble or myosin-9 siRNAs and either uninfected or infected with reovirus T3D at an MOI of 5 PFU/cell at 24 h post-transfection. Cells were incubated for 24 h. Myosin-9 and GAPDH levels were determined by immunoblotting (representative of three independent experiments). Myosin-9 silencing in uninfected and reovirus-infected cells was quantified. (TIF) [file ppat.1013597.s008.tif]

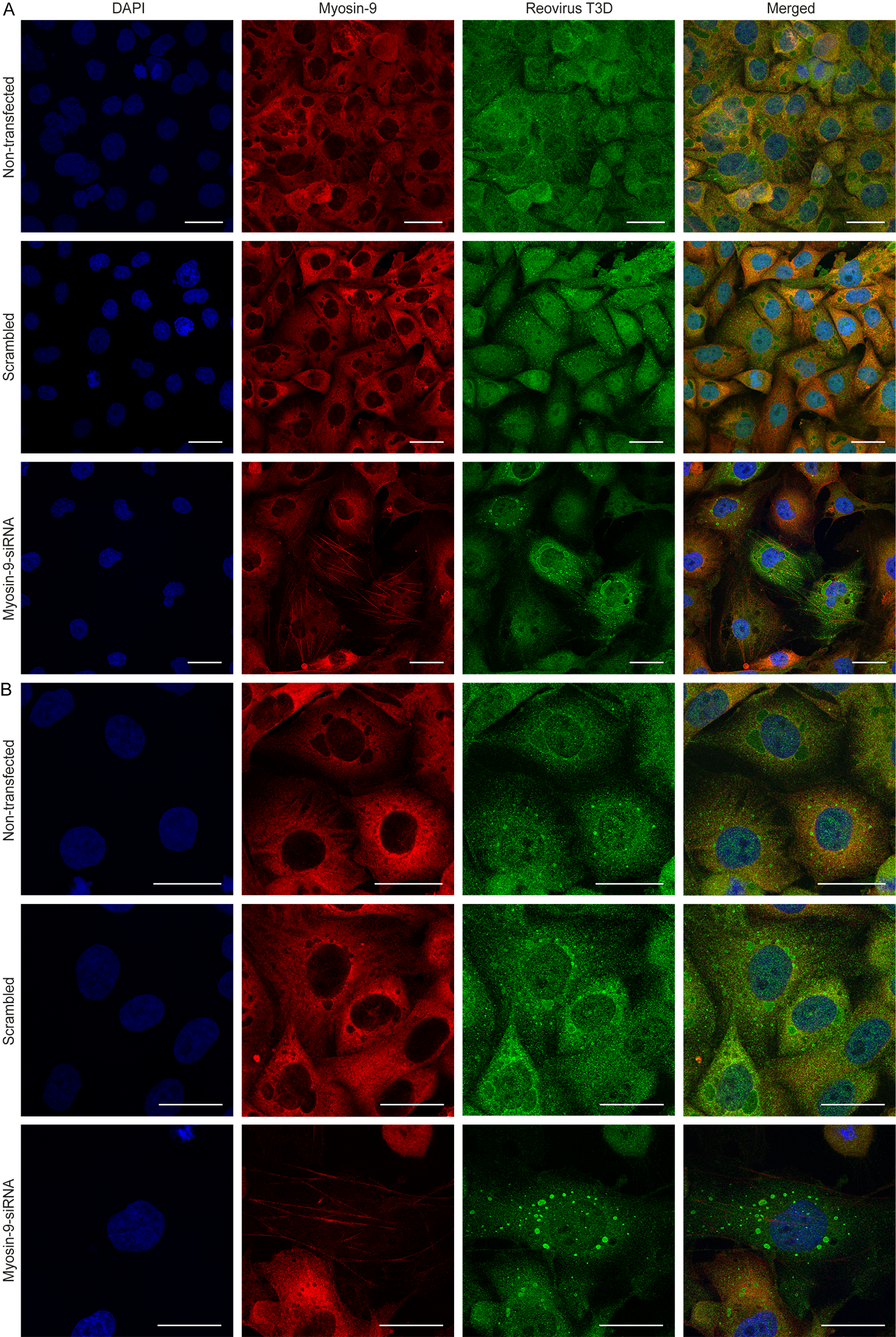

Supplement: S9 Fig — HBMECs were non-transfected or transfected with scramble or myosin-9 siRNAs and infected with reovirus T3D at an MOI of 5 PFU/cell at 24 h post-transfection. Cells were incubated for 24 h and imaged using confocal microscopy. (A and B) Immunofluorescence images of the cytoplasmic distribution of myosin-9 and reovirus in infected cells stained with antibodies specific for myosin-9 (red) and reovirus (green). Nuclei were labeled with DAPI (blue). In cells transfected with myosin-9 siRNA, the remaining myosin-9 redistributes and forms filaments in some reovirus-infected cells. The fluorescent reovirus signal concentrates primarily inside factories. Higher magnification views of selected areas are shown in B. Scale bars, 25 µm. (TIF) [file ppat.1013597.s009.tif]

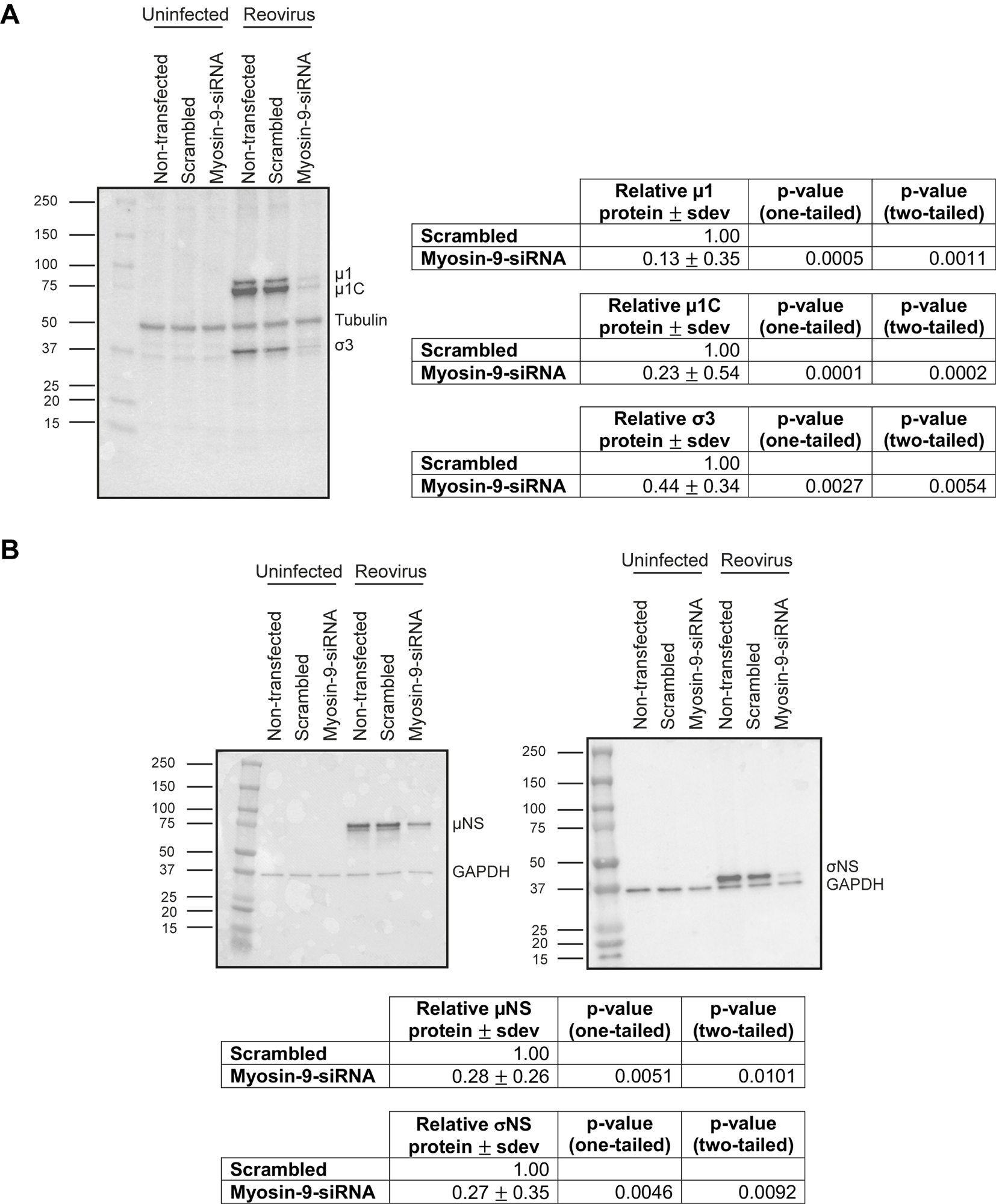

Supplement: S10 Fig — HBMECs were non-transfected or transfected with scramble or myosin-9 siRNAs and either uninfected or infected with reovirus T3D at an MOI of 5 PFU/cell at 24 h post-transfection. Cells were incubated for 24 h. (A) Reovirus structural proteins µ1, µ1C, and σ3 were detected by immunoblotting using T3D-specific antiserum. Tubulin was detected using tubulin-specific antibody. (B) Reovirus nonstructural proteins µNS and σNS were detected using chicken polyclonal and rabbit polyclonal antibodies, respectively. GAPDH was used as loading control. Immunoblots are representative of three independent experiments. Signal intensity of reovirus proteins µ1, µ1C, σ3, µNS, and σNS were quantified ± standard deviation (SD) for all the experiments. The p-values (one- and two-tailed, respectively) were calculated using student’s t-test. (TIF) [file ppat.1013597.s010.tif]

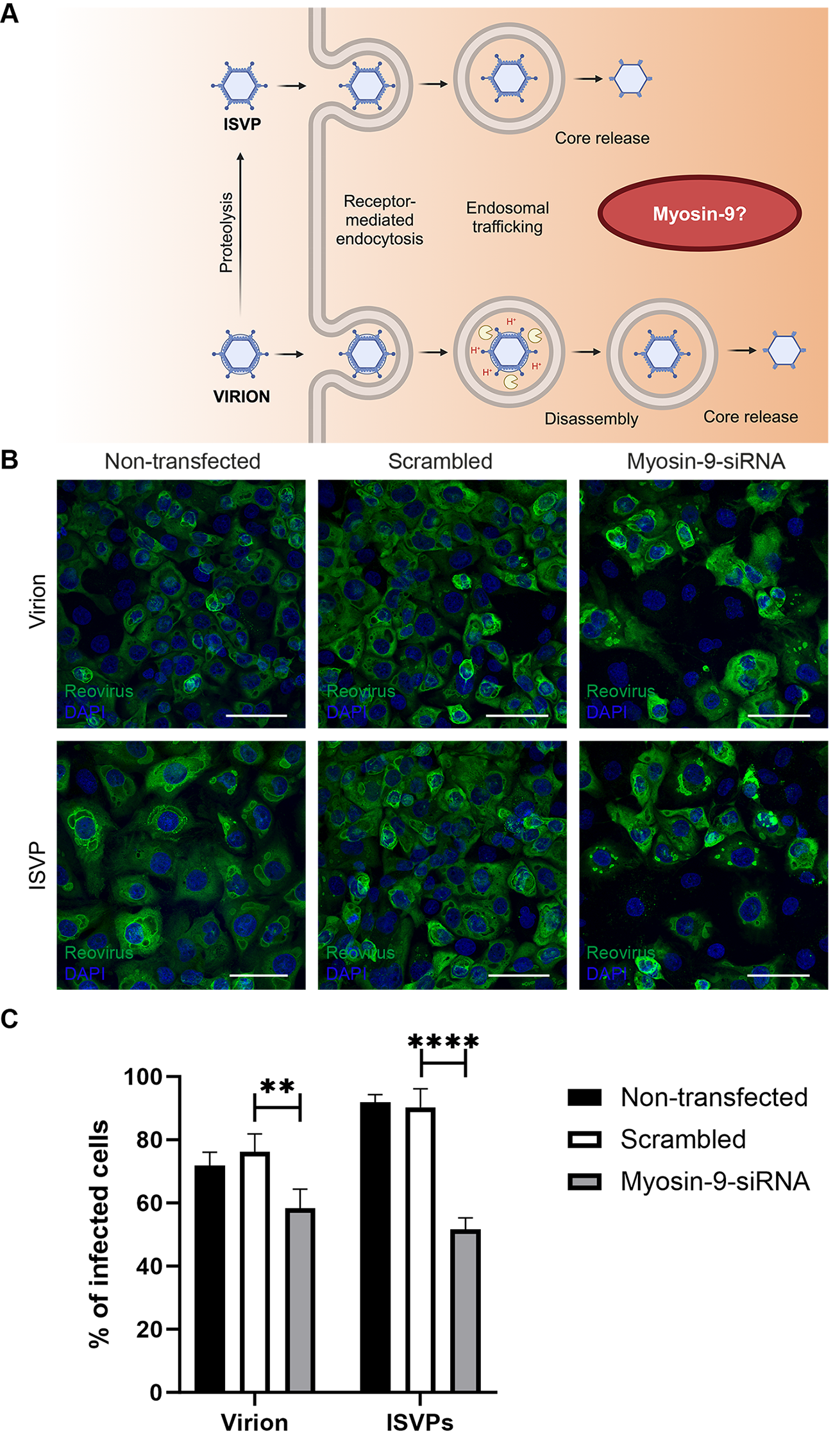

Supplement: S11 Fig — HBMECs were non-transfected or transfected with scramble or myosin-9 siRNAs and infected with reovirus T1LM1-P208S virions or infectious subvirion particles (ISVPs) at an MOI of 5 PFU/cell at 24 h post-transfection. Cells were incubated for 18 h and processed for immunofluorescence. (A) Schematic of cell entry by receptor-mediated endocytosis of virions or ISVPs. Prepared using BioRender. Tenorio, R. (2025) https://BioRender.com/oeo07s5. (B) Representative confocal micrographs of cells immunostained using a rabbit polyclonal antiserum specific for reovirus (green). Nuclei were labeled with DAPI (blue). Scale bars, 50 µm. (C) The percentage of infected cells was determined by enumerating reovirus-infected cells in immunofluorescence images and presented ± SEM of three independent experiments. Significance was determined by unpaired two-tailed student’s t-test; ** p < 0.01 and **** p < 0.0001. (TIF) [file ppat.1013597.s011.tif]

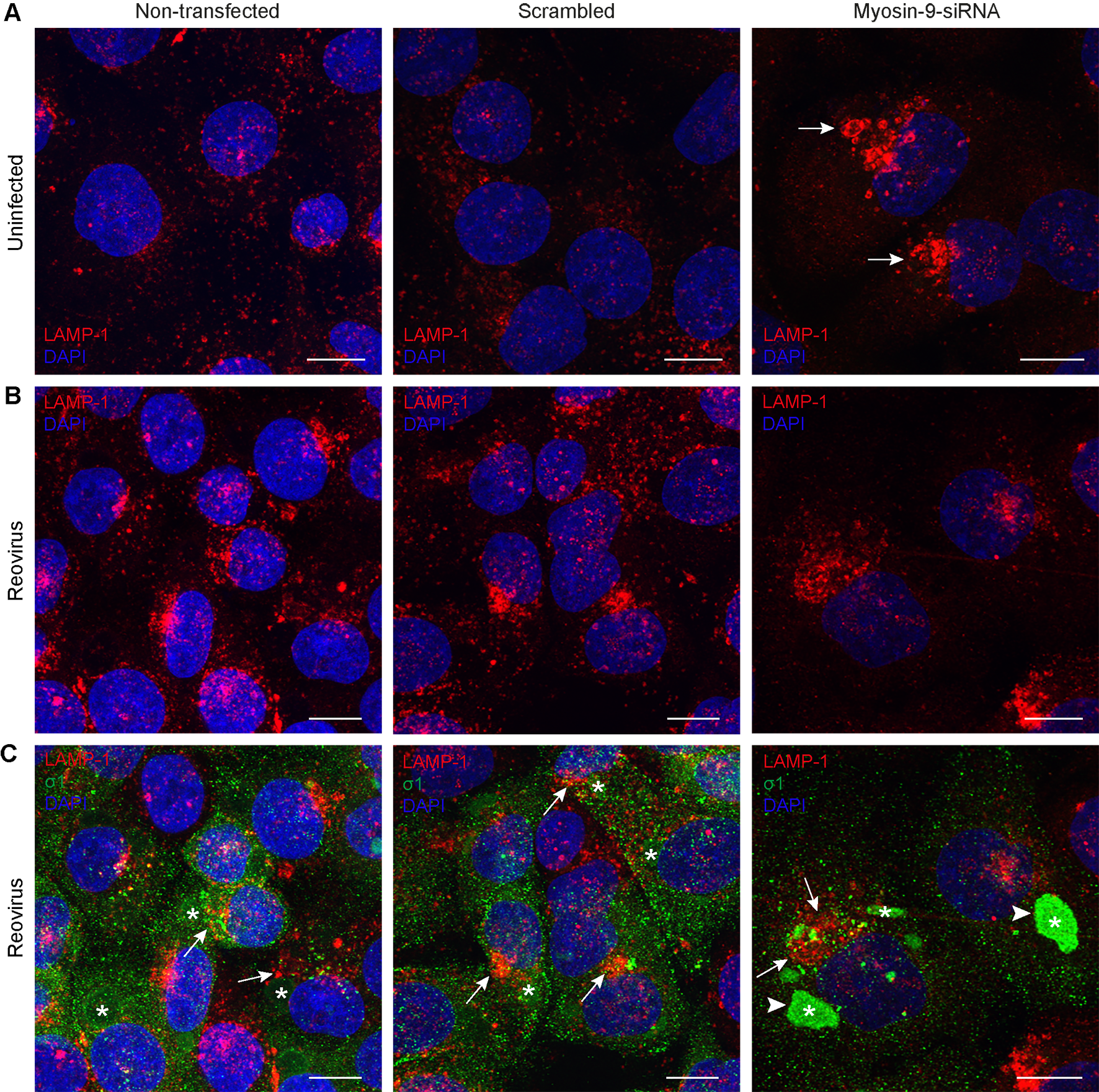

Supplement: S12 Fig — HBMECs were non-transfected or transfected with scramble or myosin-9 siRNAs and either uninfected or infected with reovirus T3D at an MOI of 5 PFU/cell at 24 h post-transfection. Cells were incubated for 24 h and imaged using confocal microscopy. Immunofluorescence images show cells stained for reovirus capsid protein σ1 (green), LAMP1 (red), and DAPI to label nuclei (blue). (A) In non-transfected or scrambled siRNA-transfected cells, LAMP1 staining (red) was distributed diffusely in the cytoplasm. In myosin-9 siRNA-transfected cells, LAMP1 staining concentrated in the perinuclear region (arrows). (B and C) In reovirus-infected cells, reovirus σ1 staining distributed in the cytoplasm in non-transfected or scrambled siRNA-transfected cells and inside reovirus factories (asterisks) in cells in which myosin-9 expression was silenced (arrowheads). Aggregated lysosomes located near the VFs are indicated with arrows. Scale bars, 10 µm. (TIF) [file ppat.1013597.s012.tif]

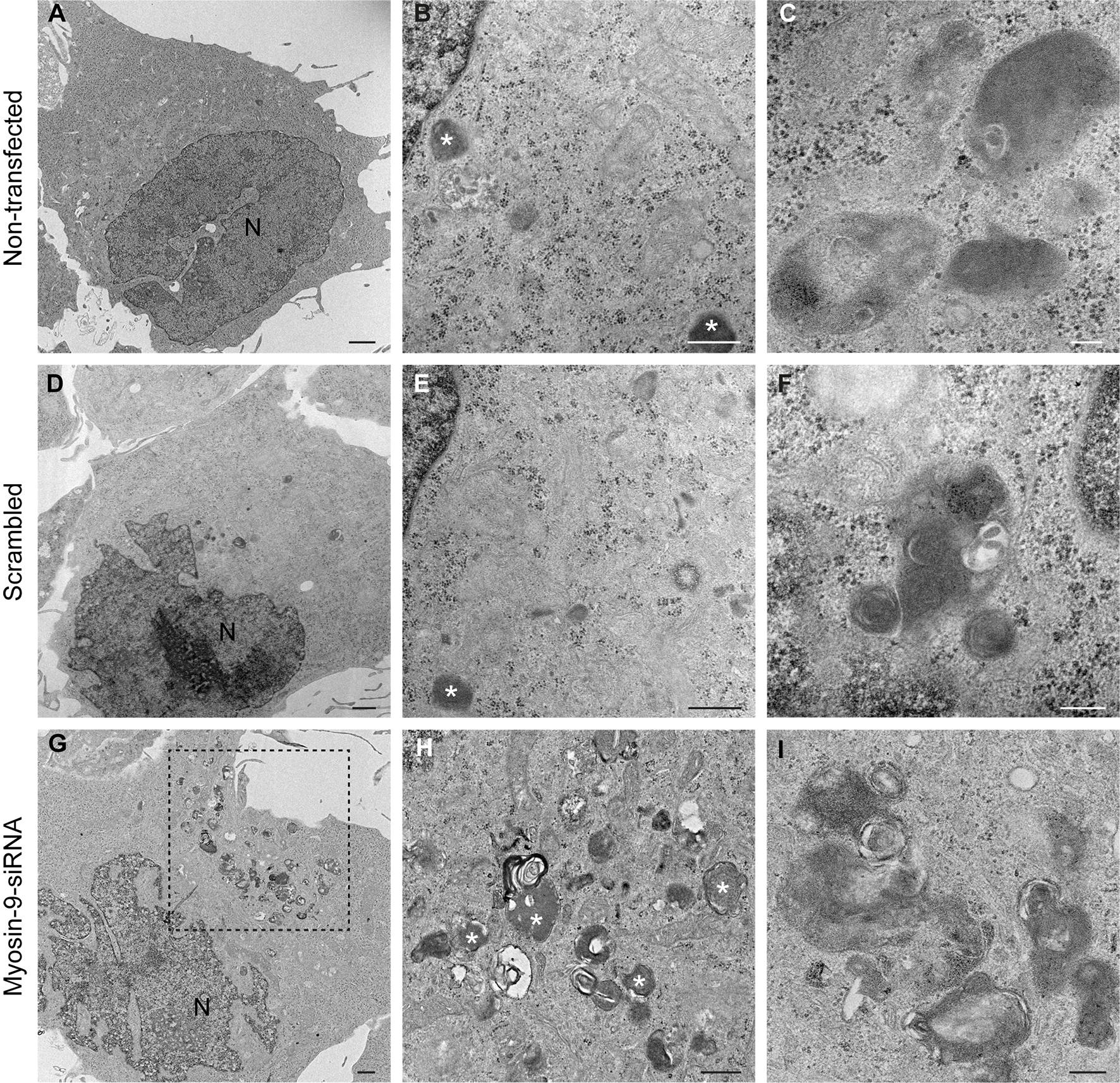

Supplement: S13 Fig — HBMECs were non-transfected or transfected with scramble or myosin-9 siRNAs. At 24 h post-transfection, cells were processed for electron microscopy. (A, D and G) Ultrathin sections of low-magnification views of cells non-transfected or transfected with scramble and myosin-9 siRNAs are shown. Lysosomes (dashed box) accumulate in a discrete area of the cytoplasm in a cell in which myosin-9 has been silenced (g). N, nucleus. Scale bars, 1 µm. (B, E and H) Higher-magnification views of the cytoplasm with lysosomes indicated (asterisks). Scale bars, 500 nm. (C, F and I) Lysosome morphology and structure. Scale bars, 200 nm. (TIF) [file ppat.1013597.s013.tif]

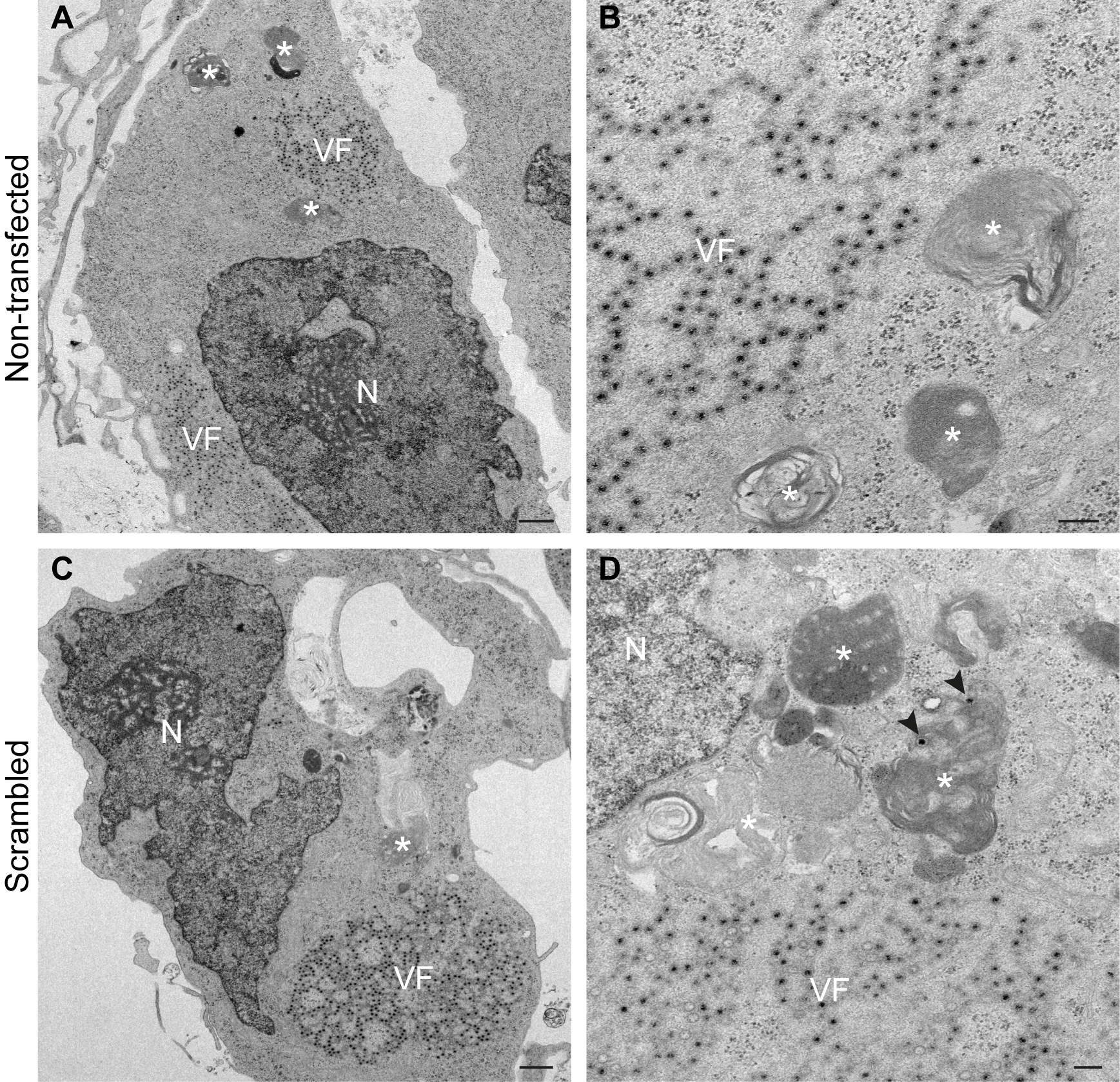

Supplement: S14 Fig — HBMECs were non-transfected or transfected with scramble siRNAs and infected with reovirus T3D at an MOI of 5 PFU/cell at 24 h post-transfection. Cells were incubated for 24 h and processed for electron microscopy. (A and C) Low-magnification views of cells non-transfected or transfected with scramble siRNA and infected with reovirus. Lysosomes (asterisks) are adjacent to VFs in the cytoplasm. Scale bars, 1 µm. (B and D) VFs and lysosomes at higher magnification. Viral particles (arrowheads) are seen inside a lysosome in a scramble siRNA-transfected cell. N, nucleus. Scale bars, 200 nm. (TIF) [file ppat.1013597.s014.tif]

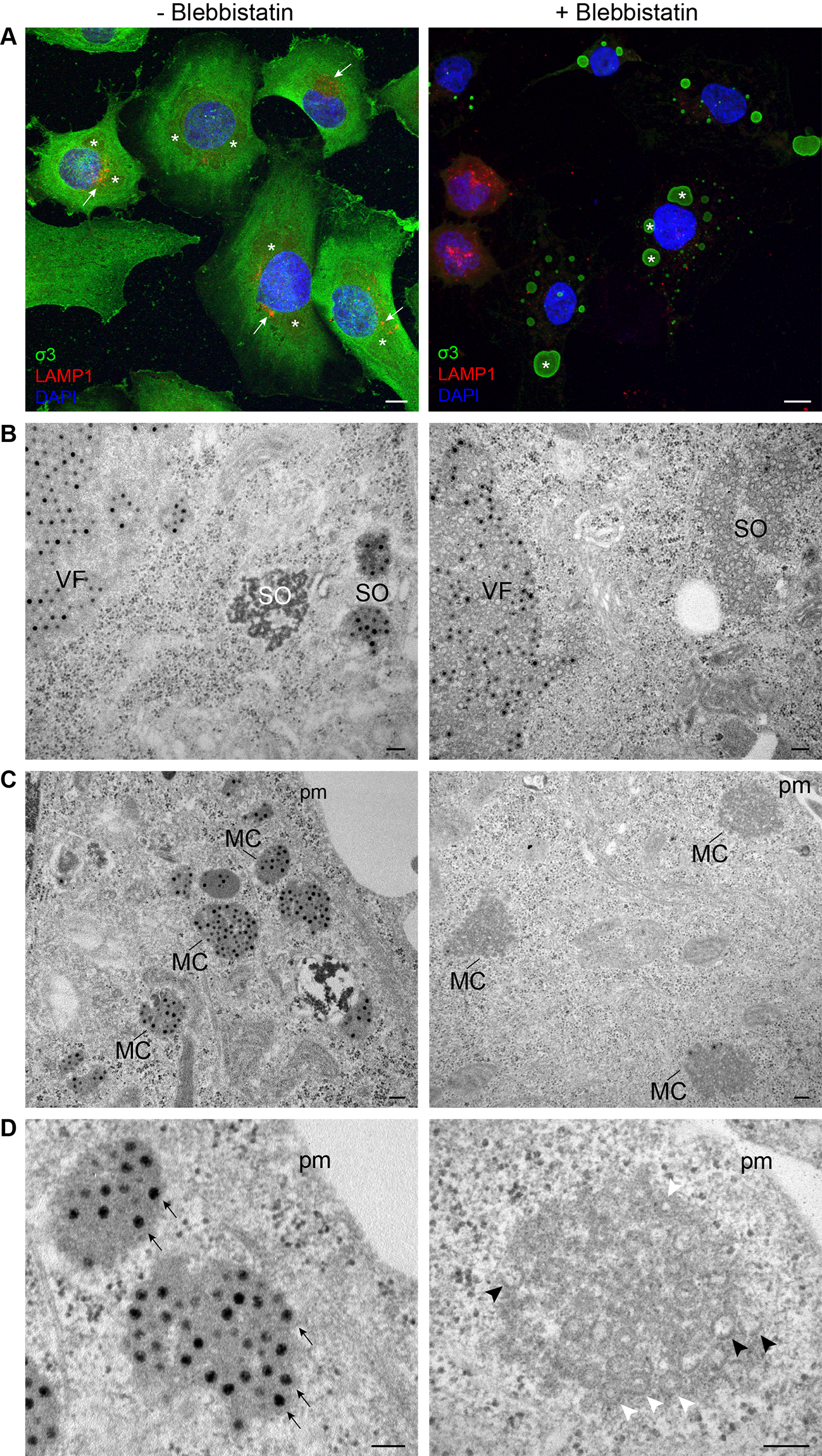

Supplement: S15 Fig — HBMECs were either uninfected or infected with reovirus T1LM1-P208S at an MOI of 1 PFU/cell. At 17 h post-infection, cells were incubated with 50 μM blebbistatin or DMSO control for 1 h and processed for confocal and electron microscopy. (A) Cells were immunostained with antibodies specific for reovirus σ3 protein (green) and LAMP1 (red). Nuclei were labeled with DAPI (blue). Images show reovirus σ3 protein distribution in reovirus-infected cells in the absence and presence of blebbistatin. In untreated infected cells, σ3 is distributed in the cytoplasm and inside VFs (asterisks). Lysosomes (white arrows) marked by LAMP1 are mostly adjacent to factories in the perinuclear region. In blebbistatin-treated cells, cytoplasmic σ3 disappears, and the protein concentrates in VFs (asterisks). The LAMP1 signal is weak in cells treated with blebbistatin. Scale bars, 10 μm. (B) EM images of infected cells in the absence (left) or presence of (right) blebbistatin treatment. In untreated cells, the SO is filled with mature virions near a VF. In blebbistatin-treated cells, the SO contains mostly immature and empty viral particles near a VF. (C) Representative examples of membranous carriers (MCs) close to the plasma membrane (pm) are shown. In untreated infected cells (left), MCs contain mature virions. In cells treated with blebbistatin (right), MCs contain mostly empty viral particles. (D) Higher-magnification images of MCs presented in C. Mature virions (arrows) in untreated cells (left) are electron dense compared with the empty capsids (black arrowheads) and immature virions (white arrowheads) observed in the cell treated with blebbistatin (right). Scale bars, 200 nm. (TIF) [file ppat.1013597.s015.tif]
