# Supplementary material for: Myosin-9 is required for lysosome-mediated nonlytic reovirus egress
Source: PLoS Pathog. 2025 Oct 14;21(10):e1013597. doi: 10.1371/journal.ppat.1013597 (PMC12543285; doi:10.1371/journal.ppat.1013597)
Supplement: S1 Table — (DOCX) [file ppat.1013597.s016.docx]

| More abundant proteins in lysosome-containing fractions purified from reovirus-infected cells | | |
| --- | --- | --- |
| Protein name | **Description** | **UniProt Accession** |
| GOLGB1 | Golgin subfamily B member 1 | Q14789 |
| KTN1 | Kinectin | Q86UP2 |
| HSPA5 | 78 kDa glucose-regulated protein | P11021 |
| PDIA4 | Protein disulfide-isomerase A4 | P13667 |
| HSP90B1 | Endoplasmin | P14625 |
| HYOU1 | Hypoxia up-regulated protein 1 | Q9Y4L1 |
| GANAB | Mannosyl-oligosaccharide alpha-1,3-glucosidase | Q14697 |
| PDIA3 | Protein disulfide-isomerase A3 | P30101 |
| RRBP1 | Ribosome-binding protein 1 | Q9P2E9 |
| ATP2A2 | Sarcoplasmic/endoplasmic reticulum calcium ATPase 2 | P16615 |

| Less abundant proteins in lysosome-containing fractions purified from reovirus-infected cells | | |
| --- | --- | --- |
| Protein name | **Description** | **UniProt Accession** |
| AHNAK | Neuroblast differentiation-associated protein AHNAK | Q09666 |
| MYH9 | Myosin heavy chain 9/10/11/14; Myosin-9 | P35579 |
| AHNAK2 | Neuroblast differentiation-associated protein ahnak | Q8IVF2 |
| FLNA | Filamin-A | P21333 |
| FLNB | Filamin-B | O75369 |
| SPTAN1 | Spectrin alpha chain, non-erythrocytic 1 | Q13813 |
| IQGAP1 | Iq motif containing gtpase activating protein 1 | P46940 |
| ACTN4 | Alpha-actinin-4 | O43707 |
| SPTBN1 | Spectrin beta chain, non-erythrocytic 1 | Q01082 |
| TLN1 | Talin-1 | Q9Y490 |

| **Proteins associated with extracellular viruses purified from HBMECs** | | |
| --- | --- | --- |
| **Protein name** | **Description** | **UniProt Accession** |
| **MYH9** | Myosin heavy chain 9/10/11/14; Myosin-9 | P35579 |
| **KRT1** | Keratin, type II cytoskeletal 1 | P04264 |
| **ACTB** | Actin, cytoplasmic 1 | P60709 |
| **KRT9** | Keratin, type I cytoskeletal 9 | P35527 |
| **ACTG1** | Actin, cytoplasmic 2 | P63261 |
| **KRT10** | Keratin, type I cytoskeletal 10 | P13645 |
| **CLTC** | Clathrin heavy chain 1 | Q00610 |
| **HSPA8** | Heat shock cognate 71 kDa protein | P11142 |
| **PKM** | Pyruvate kinase m1/2 | P14618 |
| **ANXA2** | Annexin A2 | P07355 |

| **Proteins associated with extracellular viruses purified from L929 cells** | | |
| --- | --- | --- |
| **Protein name** | **Description** | **UniProt Accession** |
| **KRT1** | Keratin, type II cytoskeletal 1 | P04264 |
| **ACTB** | Actin, cytoplasmic 1 | P60709 |
| **KRT9** | Keratin, type I cytoskeletal 9 | P35527 |
| **ACTG1** | Actin, cytoplasmic 2 | P63261 |
| **KRT10** | Keratin, type I cytoskeletal 10 | P13645 |
| **KRT2** | Keratin, type II cytoskeletal 2 epidermal | P35908 |
| **HSPA8** | Heat shock cognate 71 kDa protein | P11142 |
| **HSP90AB1** | Heat shock protein HSP 90-beta | P08238 |
| **HSP90AA1** | Heat shock protein HSP 90-alpha | P07900 |
| **TUBB3** | Tubulin beta-3 chain | Q13509 |
