# Supplementary material for: Myosin-9 is required for lysosome-mediated nonlytic reovirus egress
Source: PLoS Pathog. 2025 Oct 14;21(10):e1013597. doi: 10.1371/journal.ppat.1013597 (PMC12543285; doi:10.1371/journal.ppat.1013597)
Supplement: S3 — (DOCX) [file ppat.1013597.s019.docx]

**Striking Image**

- **Caption**

**STEM tomography image of a reovirus egress organelle showing filaments associated with virions.**

- **Credit**

Isabel Fernández de Castro and Martin Sachse

- **License:** This image is licensed under the Creative Commons Attribution 4.0 International License.

<https://creativecommons.org/licenses/by/4.0/>
